# Aromaticity Reversal Induced by Vibrations in Cyclo[16]carbon

**DOI:** 10.1021/jacs.3c10207

**Published:** 2023-12-01

**Authors:** Igor Rončević, Freddie J. Leslie, Max Rossmannek, Ivano Tavernelli, Leo Gross, Harry L. Anderson

**Affiliations:** † Department of Chemistry, Oxford University, Chemistry Research Laboratory, Oxford OX1 3TA, United Kingdom; ‡ IBM Research Europe − Zurich, Säumerstrasse 4, Rüschlikon 8803, Switzerland

## Abstract

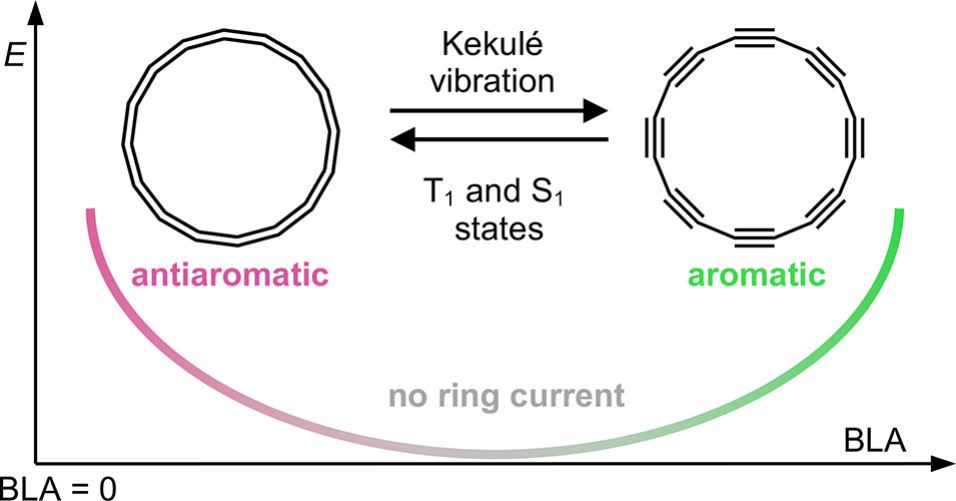

Aromaticity is typically regarded as an intrinsic property of a molecule, correlated with electron delocalization, stability, and other properties. Small variations in the molecular geometry usually result in small changes in aromaticity, in line with Hammond’s postulate. For example, introducing bond-length alternation in benzene and square cyclobutadiene by modulating the geometry along the Kekulé vibration gradually decreases the magnitude of their ring currents, making them less aromatic and less antiaromatic, respectively. A sign change in the ring current, corresponding to a reversal of aromaticity, typically requires a gross perturbation such as electronic excitation, addition or removal of two electrons, or a dramatic change in the molecular geometry. Here, we use multireference calculations to show how movement along the Kekulé vibration, which controls bond-length alternation, induces a sudden reversal in the ring current of cyclo[16]carbon, C_16_. This reversal occurs when the two orthogonal π systems of C_16_ sustain opposing currents. These results are rationalized by a Hückel model which includes bond-length alternation, and which is combined with a minimal model accounting for orbital contributions to the ring current. Finally, we successfully describe the electronic structure of C_16_ with a “divide-and-conquer” approach suitable for execution on a quantum computer.

## 1 Introduction

Aromaticity is one of the most debated concepts in chemistry, and its definition has undergone several revisions over the last few decades.^1−5^ Today, the most commonly used criterion for aromaticity is magnetic,^2^ equating the presence of a diatropic or paratropic ring current in an applied magnetic field with aromaticity or antiaromaticity, respectively. In molecules with several π systems, local and/or global currents may be present simultaneously,^6,7^ reinforcing or opposing each other. Aromaticity is a defining characteristic of a molecule, linked with reactivity, stability, HOMO–LUMO^8^ and singlet–triplet gaps,^9^ feasibility for singlet fission,^10^ diradical character,^11^ wave function coherence,^12^ and other properties.

Cyclo[*N*]carbons are all-carbon rings with two orthogonal π systems, usually with *N* electrons in each.^13^ In an analogy to annulenes, cyclocarbons with *N* = 4*n* + 2 carbon atoms can be classed as doubly aromatic (with both π systems sustaining a diatropic ring current), while *N* = 4*n* cyclocarbons are expected to be doubly antiaromatic.^14,15^ Many cyclocarbons have been found in the gas phase, but only C_10_,^16^ C_14_,^16^ C_16_,^17^ and C_18_^18^ have been structurally characterized using scanning probe microscopy.

A recent on-surface investigation of C_16_ revealed the presence of strong bond-length alternation (BLA) and confirmed that its ground state is doubly antiaromatic.^17^ Here, we investigate the variation of aromaticity with geometry in the two lowest singlets (S_0_ and S_1_), the lowest triplet (T_1_) and quintet (Q_1_) state of C_16_. Surprisingly, we find that the total ring current in the S_1_ and T_1_ states can be reversed from aromatic to antiaromatic by movement along the Kekulé vibration (∼2300 cm^–1^), i.e., by changing the amount of bond-length alternation.^19^ These aromaticity reversals require a relatively small amount of energy, in contrast with previous reports which require a change in the electronic state,^20−23^ molecular charge^24−26^ or composition,^27,28^ or involve a high-lying transition state^29,30^ or a highly strained geometry.^31^ To our knowledge, the only comparable low-energy aromaticity reversal involves a conformational equilibrium between Hückel antiaromatic and Möbius aromatic conformers in a hexaphyrin derivative.^32,33^

The simplicity and unique electronic structure of cyclocarbons makes them an interesting testing ground for theoretical methods.^13^ Quantum algorithms, which strongly benefit from execution on a quantum computer, are a promising avenue for the further development of electronic structure methods. However, their execution on current quantum devices is limited by noise and coherence time.^34,35^ Here, we exploit the orthogonality between the two π systems of C_16_ to effectively increase the active space of the quantum unitary coupled clusters singles doubles (q-UCCSD) method,^36^ which we solve variationally.^37^ This divide-and-conquer approach enables us to obtain highly accurate results that would otherwise not be feasible to compute using near-term quantum devices.

### 1.1 Ipsocentric Approach

Before discussing the electronic structure and ring current in C_16_, we briefly summarize the rules for evaluating orbital contributions to magnetic properties in the ipsocentric approach, as developed by Steiner and Fowler:^38−40^

**Figure 1:**
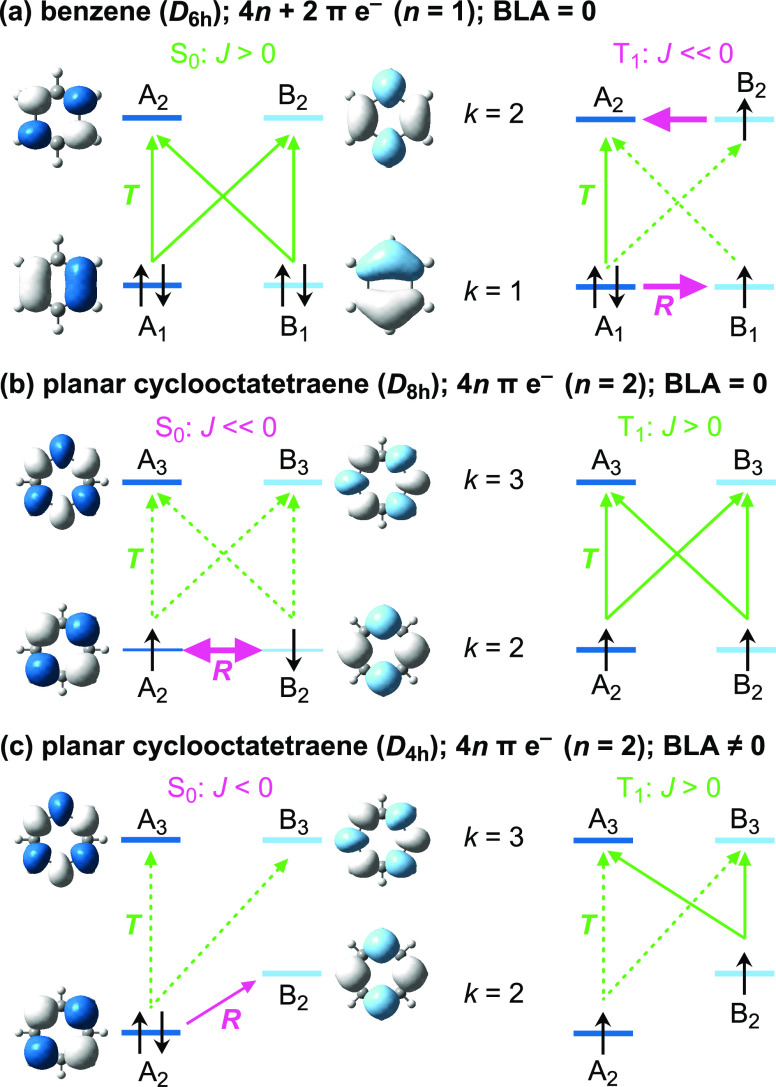
Translational (green, labeled *T*) and rotational (purple, labeled *R*) transitions in the lowest singlet (S_0_, left) and triplet (T_1_, right) state for (a) benzene, (b) planar COT with no BLA, and (c) planar COT with BLA. Full and dashed lines show relatively larger and smaller contributions to the ring current, respectively.

1. The current density *J* induced by a magnetic field (which, when integrated over the ring, gives the ring current strength, usually measured in *nA*/*T*) can be expressed as a sum of spin-allowed transitions from occupied (ψ_*s*_) to unoccupied (ψ_*t*_) orbitals. The contribution of each transition (*J*_st_) can be written as a sum of a diatropic term (*J*_*st*_^DIA^), which is associated with aromaticity, and a paratropic term (*J*_*st*_^PARA^) associated with antiaromaticity:
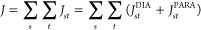
1The diatropic current, which opposes the externally applied field, is taken to be positive (*J*^DIA^ > 0), while the paratropic current, which reinforces the external field, is taken to be negative (*J*^PARA^ < 0).
2 The diatropic (aromatic) contribution of a transition from ψ_*s*_ to ψ_*t*_ to the current density (*J*_*st*_^DIA^) is determined by the orbital energy difference Δε_st_ and the translational matrix element *M*_*st*_^T^, which reflects the orbital coupling under the linear momentum operator *p̂* (represented by *T* in Figure 1):^39^
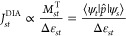
2
  In general, *M*_*st*_^T^ will be large for spatially similar orbitals differing in the number of nodal planes by one,^38^ corresponding (in planar monocyclic molecules) to a change in the angular momentum *k* by one. For example, the purely diatropic ring current in benzene can wholly be attributed to translational transitions from *k* = 1 to *k* = 2 (Figure 1a left).^40^
3 The contribution of an orbital pair to the paratropic current (*J*_*st*_^PARA^) is also modulated by Δε_*st*_ and depends on the magnitude of the rotational matrix element *M*_*st*_^R^, which couples the orbitals under the angular momentum operator *l̂* (represented by ***R*** in Figure 1):^39^
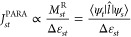
3
  For planar monocyclic molecules without BLA, *M*_*st*_^R^ will approach unity for orbital pairs related by a rotation, such as A_2_ and B_2_ in *D*_8h_ cyclooctatetraene (COT, Figure 1b; A and B correspond to sine and cosine density patterns). Such systems will have open-shell character^21,41,42^ and very large paratropic currents. Introducing BLA in planar COT (Figure 1c) lifts the degeneracy between A_2_ and B_2_, producing a closed-shell singlet, lowering the total energy and decreasing antiaromaticity.
4 From eqs 2 and 3, we can deduce that very few orbitals around the HOMO and LUMO will meaningfully contribute to the ring current. In aromatic annulenes, it is generally sufficient to consider only translational transitions with Δ*k* = 1, i.e., HOMO → LUMO. In antiaromatic annulenes, rotational transitions with Δ*k* = 0 (A_2_ ↔ B_2_ in Figure 1b,c) are responsible for most of the ring current, with weak contributions from occupied orbitals below the HOMO.^40^ We also note that this rule is valid only when the magnetic response is global, i.e., when the frontier orbitals are delocalized along the entire ring, which is valid up to ∼C_30_ in the case of even-numbered cyclocarbons.^20^

The ipsocentric approach provides an orbital-based rationalization of Baird’s rule, which predicts an aromaticity reversal in the lowest triplet (T_1_) state of annulenes compared to the S_0_ ground state.^43^ In benzene (Figure 1a right), promoting one electron to either A_2_ or B_2_ produces a very strong paratropic current, resulting in an aromaticity reversal in the T_1_ (and S_1_) excited states. On the other hand, in planar COT (Figure 1b,c) flipping an electron renders the rotational A_2_ ↔ B_2_ transitions forbidden, leading to a Baird aromatic T_1_ state. Finally, the evaluation of aromaticity in the S_1_ (and other excited states) is less straightforward due to a combination of diatropic and paratropic contributions, in agreement with the experimental observation that Baird’s rule is weaker for S_1_ than for T_1_.^44^

In the ground state (S_0_) of C_16_, the configuration of both π systems is similar to that in planar COT (Figure 1b,c). Flipping a single electron in C_16_ will result in a T_1_ state with mixed aromaticity, where one π system will be Baird aromatic and the other will remain antiaromatic (norcorrole is a similar example^45^). Flipping an electron in both π systems of C_16_ will result in a doubly Baird aromatic quintet (Q_1_) state, as shown by Fowler.^46^ We also investigate the S_1_ state in which an electron moves from one π system to the other, resulting in a pair of oppositely charged doublets.

## 2 Results and Discussion

**Figure 2:**
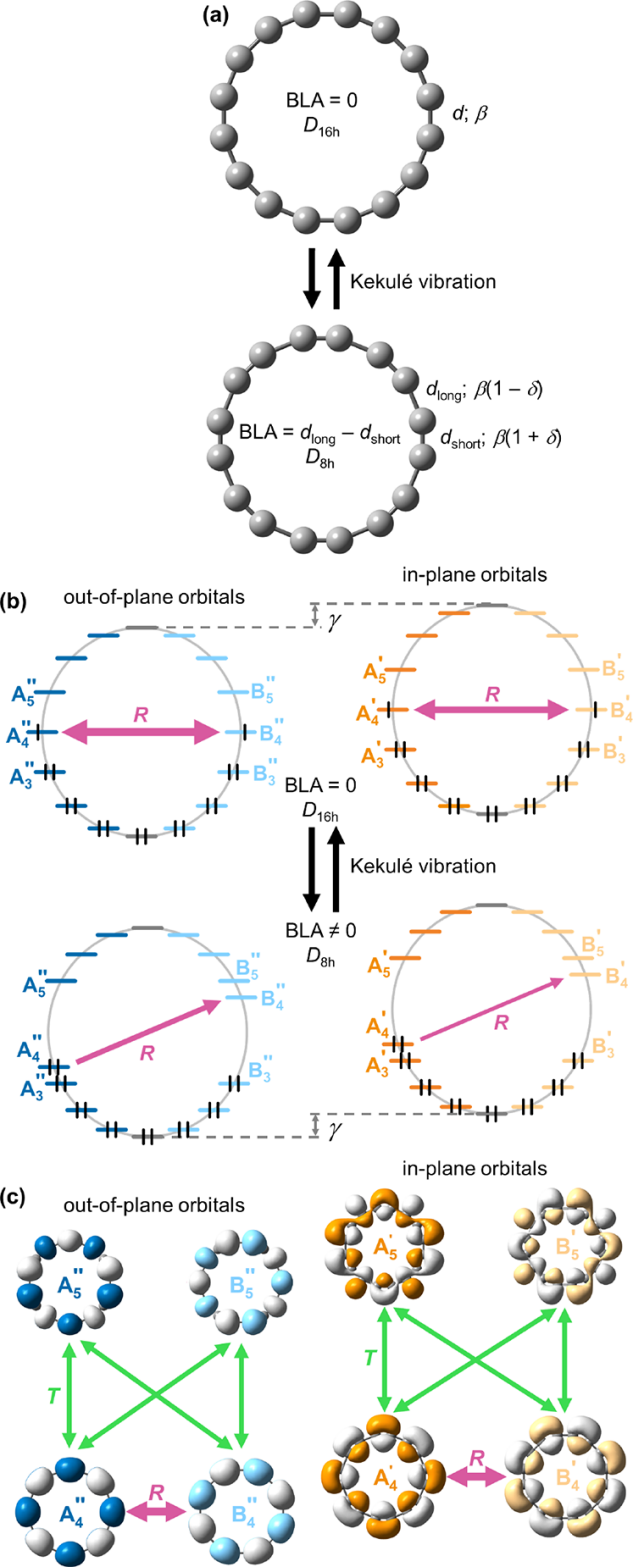
(a) BLA-inducing Kekule vibration in C16, with bond lengths *d* and nearest-neighbor couplings β denoted. Frost-Musulin diagrams (assuming β″ = β′) for C_16_ in the absence (top) and presence (bottom) of BLA, with the dominant paratropic contributions in the ground state shown in purple. Offset between the out-of-plane (blue) and in-plane (orange) π systems is denoted by γ. (b) Possible translational (***T***, green) and rotational (***R***, purple) transitions associated with *k* = 4–5 orbitals in low-lying electronic states of C_16_.

To understand how the electronic structure and magnetic properties of C_16_ change with BLA (Figure 2a), we use three different approaches: (1) a tight-binding Hückel-Heilbronner model (HHM), described in the next section; (2) the complete active space self-consistent field (CASSCF) method; and (3) density functional theory (DFT). In CASSCF calculations, all orbitals in a predefined active space are optimized, and the wave function can be written as a linear combination of many configurations, all of which contribute to the final magnetic properties. In contrast, the DFT wave function is only a single determinant, with strictly defined orbital occupations.

### 2.1 Hückel–Heilbronner model

To gain more qualitative insight in the electronic structure of C_16_, we employ a semiempirical HHM,^47^ which extends the simple Hückel model by replacing the nearest neighbor interaction energy β with an alternating pattern of β(1 + δ) and β(1 – δ), as shown in Figure 2a:
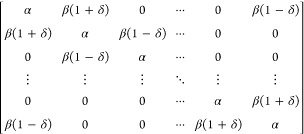
4

At δ = 0, the HHM reduces to a simple Hückel model. At 0 < δ < 1, it describes a system in which the interaction between shorter bonds is given by β(1 + δ), while longer bonds are coupled by β(1 – δ), which was shown to be a good approximation by Stanger.^48^ Previously, the HHM has been used to describe how frontier orbital energies of annulenes^19^ and azaboraheterocycles^49^ change with BLA.

C_16_ has two π systems, with out-of-plane orbitals (interaction energy β″, total energy *E*_π_^″^) offset by γ relative to in-plane orbitals (interaction energy β’, total energy *E*_π_^′^). Using HHM, we can find the π orbital energies of C_16_ by solving (4) twice, allowing for β″ ≠ β′, as shown in Figure 2b. The total energy *E*_TOT_^HHM^ can be obtained by adding the sigma contribution *a*_σ_δ^2^ (approximated as a parabola with the minimum at δ = 0),^47^ to the energies of the two π systems (*E*_π_^″^ and *E*_π_′):
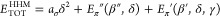
5

In C_16_ without BLA, the frontier out-of-plane orbitals (A_4_” and B_4_”; Figure 2b top) are energy degenerate and slightly (γ = 0.1 eV) lower in energy than their in-plane counterparts (A_4_′ and B_4_′), resulting in an unstable double open shell singlet,^17^ in analogy to *D*_4h_ cyclobutadiene^50^ or *D*_8h_ COT.^21,23,42^

Introducing BLA lowers the energies of A_4_″ and A_4_′ by 2β″δ and 2β′’δ (and raises the energies of B_4_″ and B_4_′ by the same value), resulting in a closed-shell doubly antiaromatic configuration. More generally, the HHM predicts that the introduction of BLA lowers the π energy of both 4*n* and 4*n* + 2 systems, with the effect being smaller in 4*n* + 2 than 4*n* systems with equal *n* and increasing with *n* (details in SI). Therefore, a π-conjugated molecule will have no BLA if its σ contribution outweighs the π contribution, which usually occurs in 4*n* + 2 molecules with small *n* (e.g., benzene), in agreement with Shaik’s interpretation based on valence bond theory.^51^ A more rigorous discussion on the distortivity of π-conjugated systems can be found in refs (52, 53, and 54).

We extend the HHM to excited states by promoting electrons to unoccupied orbitals and adding a state-specific offset *E*_xc_ to the energy of the newly occupied orbitals, in order to account for orbital relaxation and exchange coupling; in state-averaged fashion, *a*_σ_, β″, and β′ are kept equal in all states (and at all values of BLA).

Two limitations of the HHM are that it is valid only at small BLA (δ ≈ 0) and that it only accounts for the changes in orbital energies, meaning that it cannot identify a possible switch from the global to a local current.

### 2.2 Minimal Ipsocentric Model for C_16_

To provide a direct relation between aromaticity and orbital energies, we employ a minimal ipsocentric model in which matrix elements in eqs 2 and 3 are replaced by orbital-independent parameters, and only a few transitions are counted. In this approach, the total ring current of C_16_ is calculated as the sum of its out-of-plane and in-plane components, which are given by
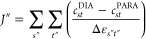
6
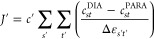
7

In eqs 6 and 7, *c*_*st*_^DIA^ has a constant positive value for all transitions with Δ*k* = 1 (e.g., A_4_” → A_5_”), while *c*_*st*_^PARA^ has a constant positive value for all Δ*k* = 0 transitions; both are zero for all other transitions. The third parameter in the model, *c*′, relates the in-plane and the out-of-plane matrix elements (more generally, it relates the currents produced in different π systems by an orbital pair with the same Δε_*st*_). *c*_*st*_^DIA^, *c*_*st*_^PARA^, and *c*′ are assumed to be independent of BLA and electronic state; for a specific molecule, they reflect how much current is induced by a single transition between orbitals separated by one unit of energy.

Within this minimal model, only orbitals with *k* = 3–5 contribute to the ring current of C_16_ (as only Δ*k* = 0 and Δ*k* = 1 transitions are counted). For simplicity, we will focus on transitions involving *k* = 4 and *k* = 5 orbitals shown in Figure 2c, as the diatropic contribution of *k* = 3 → *k* = 4 transitions changes in the same manner as the contribution from *k* = 4 → *k* = 5 transitions.

In principle, the three parameters *c*_*st*_^DIA^, *c*_*st*_^PARA^, and *c*′ (or two, in the case of an annulene) can be obtained by only three (or two) calculations. In the next section, we use a more sophisticated approach based on CASSCF ring currents and HHM orbitals but note that similar values of *c*_*st*_^DIA^, *c*_*st*_^PARA^, and *c*′ can be obtained by only three DFT calculations (see SI).

### 2.3 CASSCF and HMM results

We now investigate the variation of the ring current with BLA in the S_0_, Q_1_, T_1_, and S_1_ states of C_16_ at a series of 11 *D*_8h_ geometries with different values of BLA. The considered geometries are interpolated between the ground-state minimum previously found by NEVPT2^17^ (BLA = 11.4 pm, ring radius *r* = 3.33 Å) and the minimum-energy *D*_16h_ geometry at the same level of theory (BLA = 0 pm, *r* = 3.32 Å); results for geometries extended to BLA up to 16 pm, which roughly corresponds to the CASSCF minimum at *r* = 3.32 Å, are given in Figure 8.

CASSCF calculations include 12 electrons in 12 orbitals with *k* = 3–5 in the active space, capturing all Δ*k* = 0 and Δ*k* = 1 transitions. These CASSCF(12,12) energies (*E*_CAS_) and ring curents (*J*_CAS_), calculated from nucleus independent chemical shifts (NICS(2)_*zz*_) are used to obtain the optimal parameters for the HHM and the minimal ipsocentric model (*E*_HHM_, *J*_HHM_). In both cases, *J* is expressed relative to the benzene ring current (*J*_ref_ = 12 nA/T).

**Figure 3:**
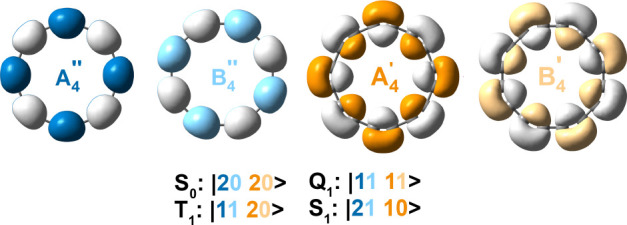
Most important configurations in the investigated electronic states of C_16_.

In the following section, different configurations are named according to the occupancies of their four *k* = 4 orbitals^17^ (Figure 3), e.g., S_0_ at large BLA (Figure 2a bottom) can be written as |20 20>. For each electronic state, the magnetic couplings in the most relevant configuration are described. The effect of dynamic correlation is evaluated by comparing the CASSCF and QD-NEVPT2^55^ wave function composition, as well as by density functional theory (DFT) calculations (ωB97XD/def2-TZVP, Figure S1 in SI).

### 2.4 S_0_ State

**Figure 4:**
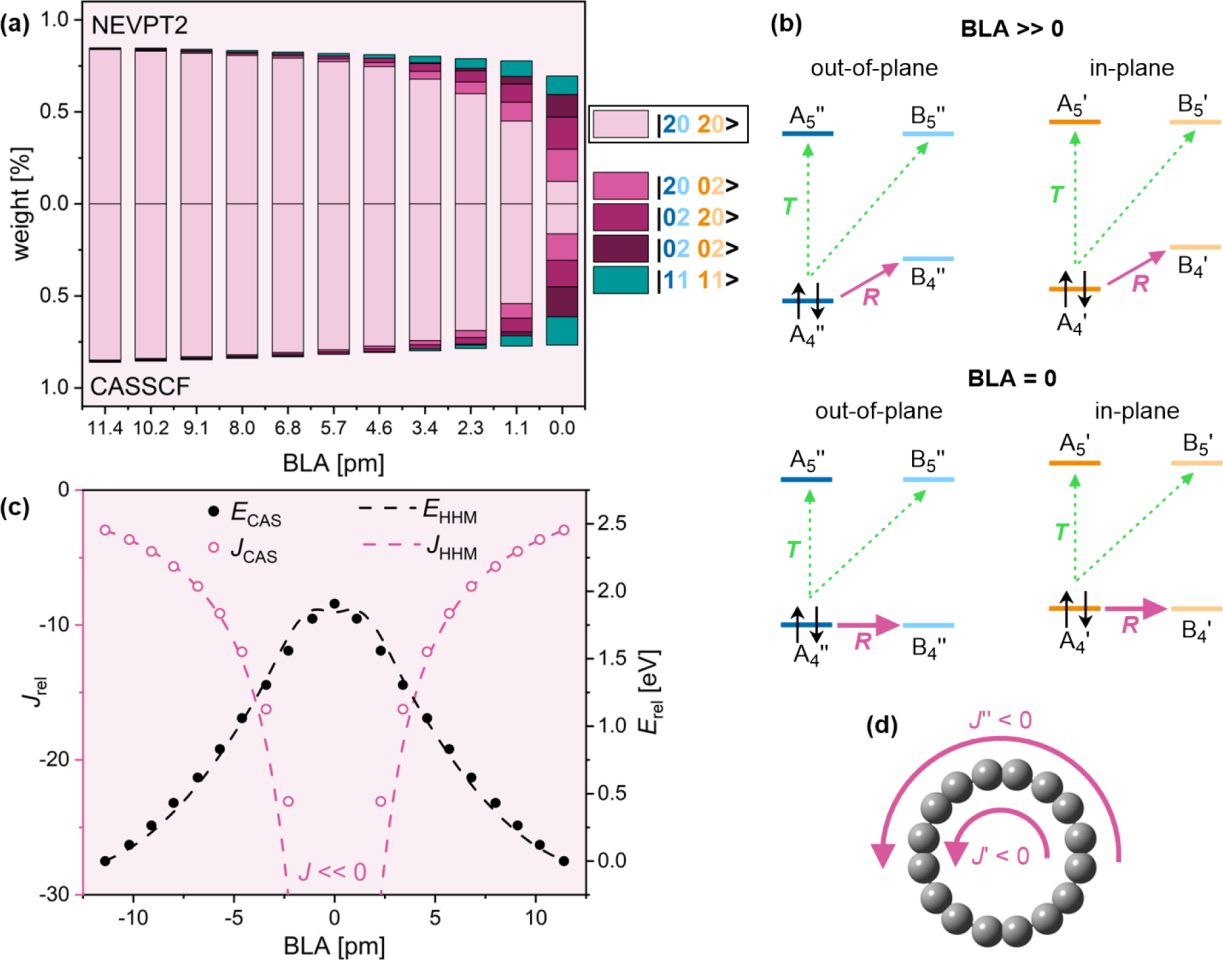
Aromaticity of C_16_ in the ground (S_0_) state. (a) Wave function composition at different BLA, as calculated by QD-NEVPT2 (top) and CASSCF (bottom). (b) Translational (green) and rotational (purple) transitions in *k* = 4–5 orbitals at large and zero BLA in the |20 20> configuration. Full and dashed lines show larger and smaller contributions to the ring current. (c) Total ring current and relative energy at different BLA calculated by CASSCF (circles) and the HHM (dashed lines). (d) In-plane (*J*′) and out-of-plane (*J*″) contributions to the total ring current.

In the ground state, the dominant configuration at virtually all nonzero BLA values is the doubly antiaromatic |20 20> (Figure 4a,d). In this configuration, A_4_ orbitals in both π systems are doubly occupied and their B_4_ counterparts are doubly unoccupied, leading to A_4_″→ B_4_″ and A_4_′ → B_4_′ rotational transitions (Figure 4b).

Decreasing BLA reduces the energy difference between A_4_ and B_4_, which rapidly increases antiaromaticity and electronic energy (Figure 4c; we define *E*_rel_ = 0 for BLA = 11.4 pm). Despite a strong multireference character at low BLA (Figure 4a), HHM reproduces the CASSCF results well (Figure 4c), with low mean absolute errors for energy MAE_*E*_ = 0.05 eV and ring current (MAE_*J*_ = 1.0).

There is a complete absence of the doubly aromatic |22 00> configuration, even at zero BLA, which is a consequence of a small separation γ between the two π systems.^17^ This is in contrast with single-reference methods and HHM, which incorrectly predict a |2200> ground state for the BLA = 0 configuration.

### 2.5 Q_1_ State

**Figure 5:**
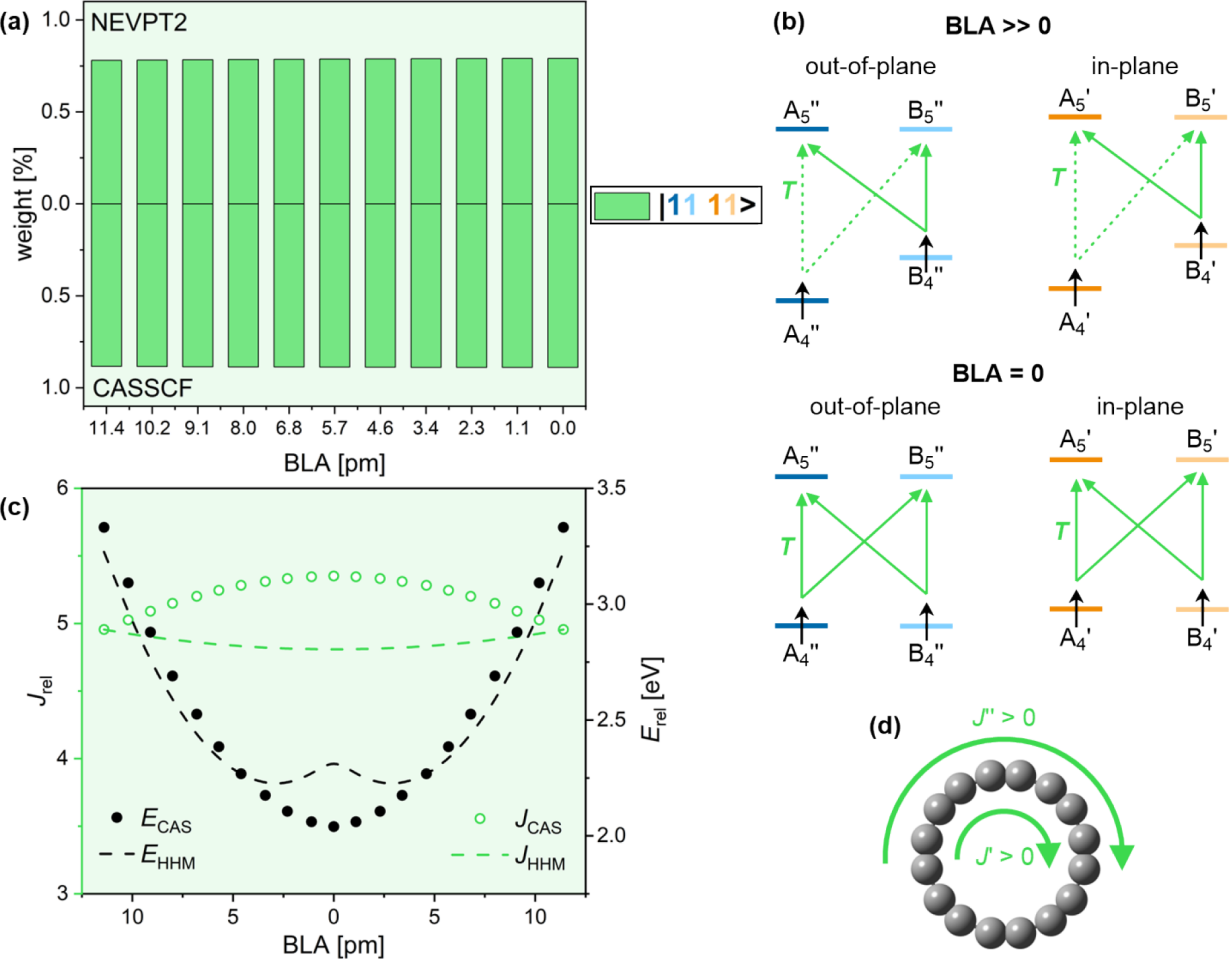
Aromaticity of C_16_ in the lowest quintet (Q_1_) state. (a) Wave function composition at different BLA, as calculated by QD-NEVPT2 (top) and CASSCF (bottom). (b) Translational (green) and rotational (purple) transitions in *k* = 4–5 orbitals at large and zero BLA in the |11 11> configuration. Full and dashed lines show larger and smaller contributions to the ring current. (c) Total ring current and relative energy at different BLA calculated by CASSCF (circles) and the HHM (dashed lines). (d) In-plane (*J*′) and out-of-plane (*J*″) contributions to the total ring current.

The major configuration in the lowest quintet state is ^5^|11 11>, in which the four *k* = 4 orbitals are singly occupied by four same-spin electrons. This wave function composition remains largely unaffected by changes in BLA (Figure 5a). No rotational transitions are allowed, leading to double aromaticity (Figure 5b,d).^46^

The Q_1_ ring current does not change much (∼10%) with BLA, as the increase in the energy of the A_4_ orbitals is offset by a decrease in B_4_ energies. At BLA = 0, the energy is minimized and the ring current is maximized (Figure 5c), which is a complete reversal of the result obtained for the doubly antiaromatic S_0_ (cf. Figures 4c and 5c). A very similar result is obtained with DFT (Figure S1b).

The HHM closely reproduces the electronic energies obtained by CASSSCF (Figure 5c; MAE_*E*_ = 0.12 eV). The ring current fit is of similar accuracy to S_0_ (MAE_*J*_ = 0.9), but it is limited by the inability of the minimal ipsocentric model to describe variation not driven by changes in orbital energies.

### 2.6 T_1_ State

**Figure 6:**
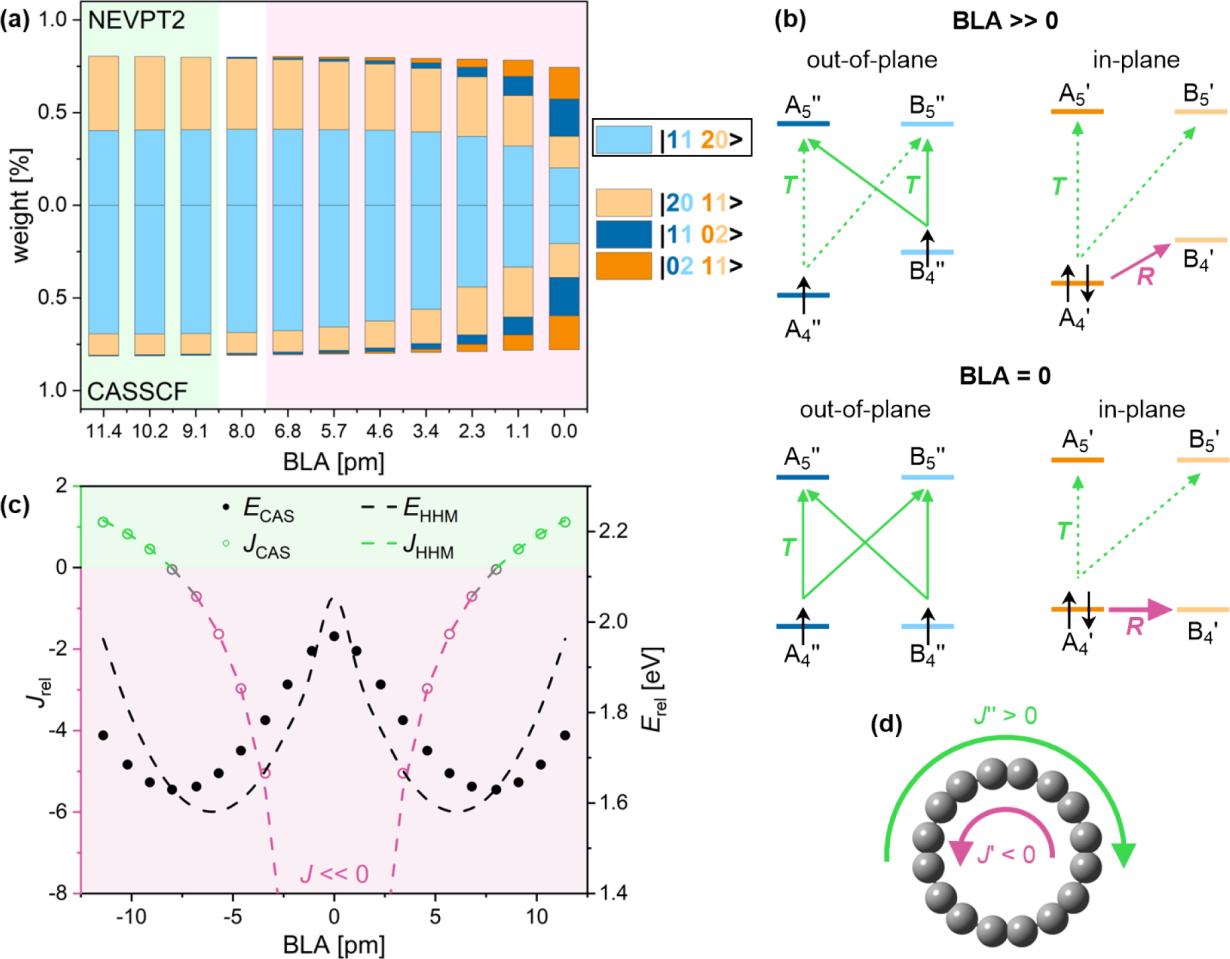
Aromaticity of C_16_ in the lowest triplet (T_1_) state. (a) Wave function composition at different BLA, as calculated by QD-NEVPT2 (top) and CASSCF (bottom). (b) Translational (green) and rotational (purple) transitions in *k* = 4–5 orbitals at large and zero BLA in the |11 20> configuration. Full and dashed lines show larger and smaller contributions to the ring current. (c) Total ring current and relative energy at different BLA calculated by CASSCF (circles) and the HHM (dashed lines). (d) In-plane (*J*′) and out-of-plane (*J*″) contributions to the total ring current.

In a naïve single-reference picture, the lowest lying triplet state (T_1_) might be obtained by changing the spin of a single electron in the S_0_ |20 20> configuration. For example, flipping the spin of an electron in the out-of-plane π system results in the |11 20> configuration, which is predicted by both CASSCF and NEVPT2 to have the largest contribution to the multireference wave function (Figure 6a).

In |11 20>, the out-of-plane |11> π system produces a diatropic current, which does not depend much on the BLA (analogously to Q_1_; Figure 6b left), while the paratropic current produced by the in-plane |20> π system strongly increases with decreasing BLA (analogously to S_0_; Figure 6b). At this point, very small changes in energy (e.g., 70 meV in either direction, which corresponds to the expectation value of the value of position along the Kekulé vibration at 0 K) result in drastic changes in the ring current (from +0.9 to −2.3 relative to benzene). Unless a measurement with femtosecond resolution can be made, we will therefore observe an average paratropic current (around −1.4 relative to benzene); however, this result illustrates the extreme variation in the magnetic susceptibility with small changes in energy and geometry.

The HHM qualitatively recovers the variation of energy with BLA, and reproduces the ring current changes with relatively high accuracy (Figure 6c; MAE_*E*_ = 0.09 eV; MAE_*J*_ = 0.3). The contribution of other notable configurations, such as |20 11> (in which an in-plane electron was flipped), is analogous to |11 20>, with a combination of paratropic and diatropic contributions. This is demonstrated by DFT, which predicts similar variation *J* with BLA for both cases (Figure S1c), in qualitative agreement with CASSCF.

### 2.7 S_1_ State

Unlike all previously considered states, the first excited singlet (S_1_) is antisymmetric with respect to the reflection of the molecular plane. This means it consists of configurations with an odd number of electrons in both π systems and can be described as a pair of doublets, with, e.g., 17 in-plane and 15 out-of-plane, or 15 in-plane and 17 out-of-plane electrons.

**Figure 7:**
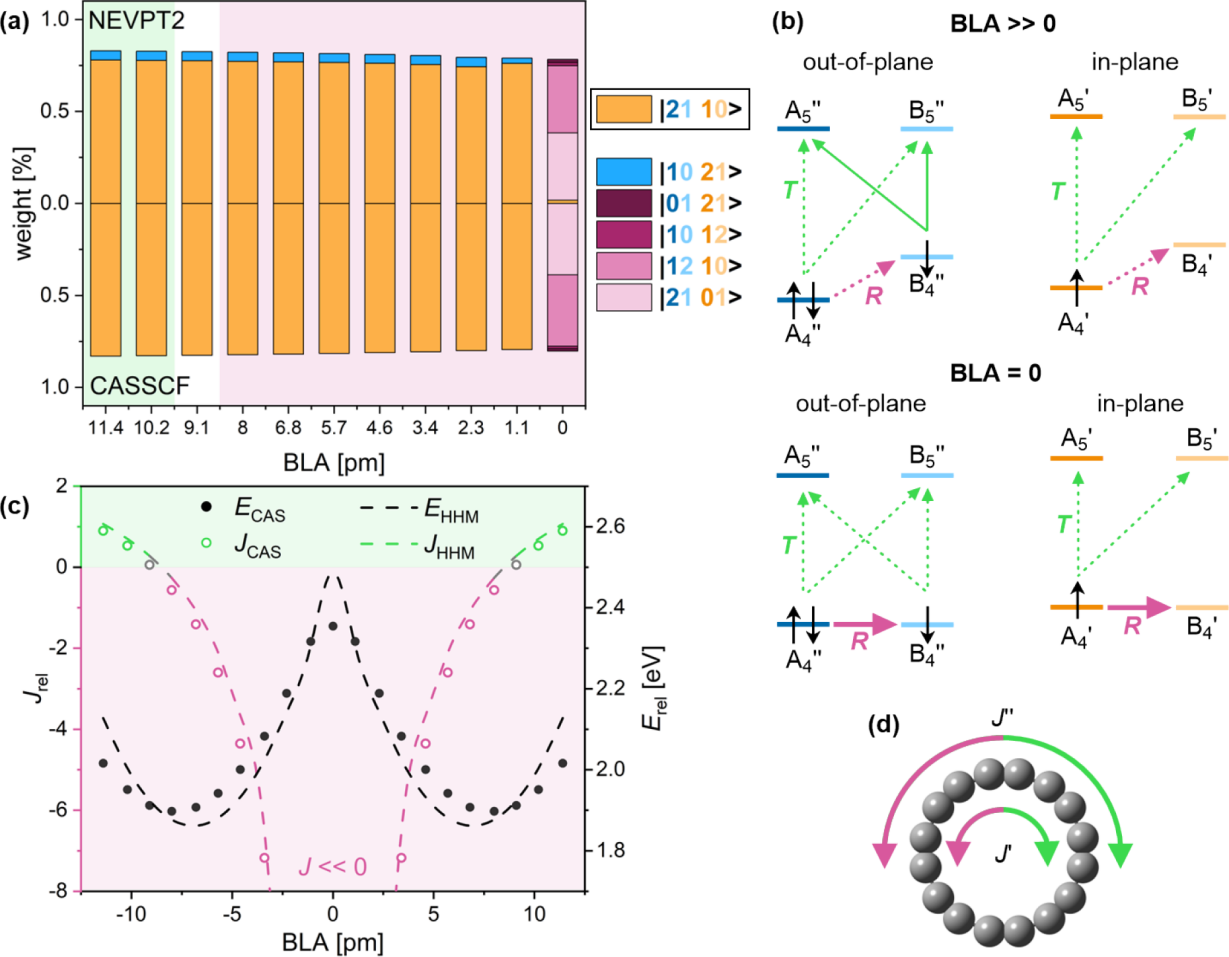
Aromaticity of C_16_ in the first excited singlet (S1) state. (a) Wave function composition at different BLA, as calculated by QD-NEVPT2 (top) and CASSCF (bottom). (b) Translational (green) and rotational (purple) transitions in *k* = 4–5 orbitals at large and zero BLA in the |21 10> configuration. Full and dashed lines show larger and smaller contributions to the ring current. (c) Total ring current and relative energy at different BLA calculated by CASSCF (circles) and the HHM (dashed lines). (d) In-plane (*J*′) and out-of-plane (*J*″) contributions to the total ring current.

At nonzero BLA, both CASSCF and NEVPT2 predict |21 10>, which has 17 out-of-plane and 15 in-plane electrons, as the dominant configuration (Figure 7a). In |21 10>, a combination of rotational and translational transitions is present both π systems (Figure 7b), resulting in mixed aromaticity (Figure 7d). At large BLA (>9.1 pm), translational contributions are stronger, resulting in an overall diatropic current. At smaller (<9.1 pm) BLA, the energy difference between A_4_ and B_4_ orbitals sufficiently shrinks to induce a reversal of the ring current (Figure 7c).

The similarity in the variation of energy and ring current in the T_1_ (Figure 6) and S_1_ (Figure 7) states can be rationalized by noting that their energies are approximately related by *E*_S1_ = *E*_T1_ + γ – *E*_EX_, where γ is the offset between the in-plane and out-of-plane orbitals and *E*_EX_ is the exchange interaction. Analogously, ring currents in T_1_ and S_1_ are similar because the number of diatropic and paratropic transitions in both is equal (cf. Figures 6b and 7b). At BLA = 9.1, the ring current in S_1_ is slightly more sensitive to small changes in energy, with a 70 meV variation in either direction changing *J* from +0.8 to −3.5.

The HHM fit to CASSCF energies and ring current is comparable to that of other states (MAE_*E*_ = 0.06 eV; MAE_*J*_ = 0.6), indicating that the HHM and the minimal ipsocentric approach can be extended to excited states.

### 2.8 Qualitative Modeling

**Figure 8:**
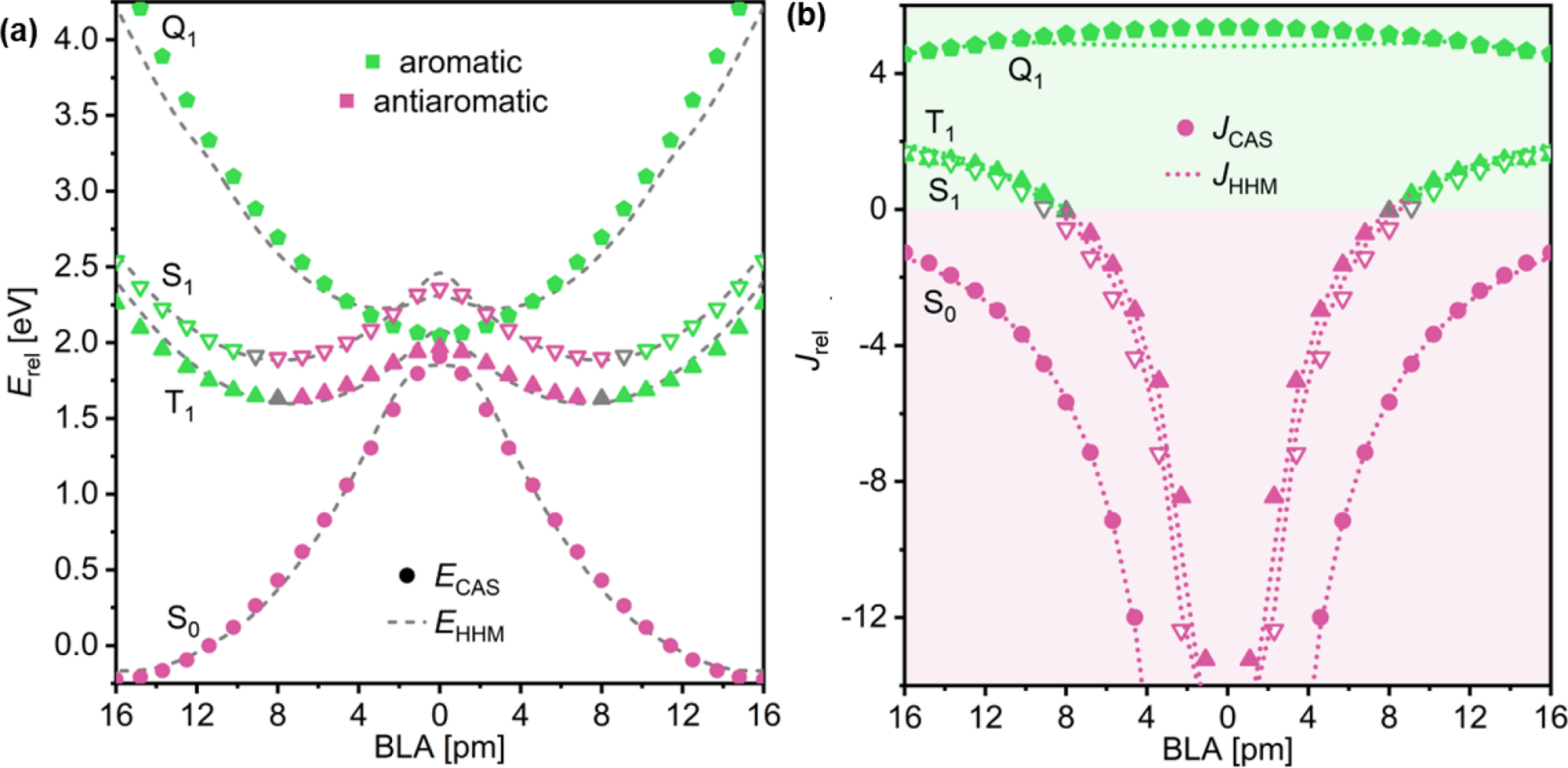
Comparison between (a) energies and (b) ring current strengths (b) predicted by CASSCF (large symbols) and the HHM (dotted lines). Circles correspond to the S_0_ state, hollow triangles correspond to S_1_, full triangles correspond to T_1_, and pentagons correspond to Q_1_. Aromatic and antiaromatic ring currents are shown in green and purple, respectively.

The state-averaged HHM is successful in describing the variation of energy with BLA in the four lowest-lying electronic states of C_16_ (Figure 8a), with MAE_*E*_ = 0.09 eV. The HHM orbital separation γ is 0.2 eV, in good agreement with the γ = 0.1 eV value previously found by DFT.^17^ We also obtain a ∼38% stronger Hückel coupling between the out-of-plane (β” = 6.1 eV) than the in-plane (β′ = 4.4 eV) orbitals, indicating stronger overlap in the out-of-plane π system.

The frontier orbital ipsocentric approach accurately reproduces the aromaticity switching predicted by CASSCF, remaining remarkably robust through a large range of ring current strengths (Figure 8b), with a mean relative error of 11.9%. Its success validates the approximation that variation in the magnetic response can be recovered purely through the change in frontier orbital energies. The coupling constants we obtain are *c*^DIA^ = 6.05 *J*_ref_/eV and *c*^PARA^ = 8.05 *J*_ref_/eV, providing a simple, direct link between orbital energy and aromaticity through eqs 6 and 7. In agreement with previous work,^20,46,56^ our minimal ipsocentric approach finds a weaker magnetic coupling in the in-plane π system (*c*′ = 0.55) relative to the out-of-plane system. Further details on the HHM and the minimal ipsocentric approach are given in the SI.

While we have only focused on the effect of BLA, aromaticity reversals may occur with any distortion that causes a stronger response in the orbital energies than in their spatial overlap. For example, bond-angle alternation (BAA) could be described by adding a β_BAA_(1 ± δ_BAA_) term to the in-plane π system. In cases where no significant open-shell character is present, the minimal ipsocentric approach could employ orbital energies calculated by DFT, making the calculation of coupling constants that relate aromaticity and frontier orbital energy very simple.

The addition of dynamic correlation does not seem to have a significant effect on the ring currents: DFT also predicts an aromaticity reversal in T_1_ (Figure S1c), and perturbatively including coupling with unoccupied orbitals outside the active space (QD-NEVPT2) does not significantly change the composition (Figures 3a–6a) of the CASSCF wave function. This is in agreement with previous work comparing the performance of HF and DFT, which concluded that the computed current depends more strongly on the used geometry than on the level of theory.^57,58^

### 2.9 Divide-and-Conquer Approach

Due to their orthogonality, the two π systems in cyclocarbons are usually considered separately.^20,46,56^ This suggests that one could approximate a 12,12 active space, which is used throughout this work, with a combination of in-plane and out-of-plane 6,6 active spaces. Here, we compute the energy of the two 6,6 subspaces (*E*′_qUCCSD_ and *E*″_qUCCSD_) using the q-UCCSD* method, which includes all single and double excitations between all spin–orbitals in the active space (further details are given in the SI). The total energy is calculated according to
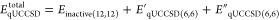
8where *E*_inactive(12,12)_ is the complete active space configuration interaction inactive energy, which can be obtained efficiently without solving the entire active space. It should be noted that in each of the two q-UCCSD(6,6) calculations, the correlated π-system feels the static orbitals of the noncorrelated π-system, i.e., the two π-system systems remain coupled at a mean-field level.

**Figure 9:**
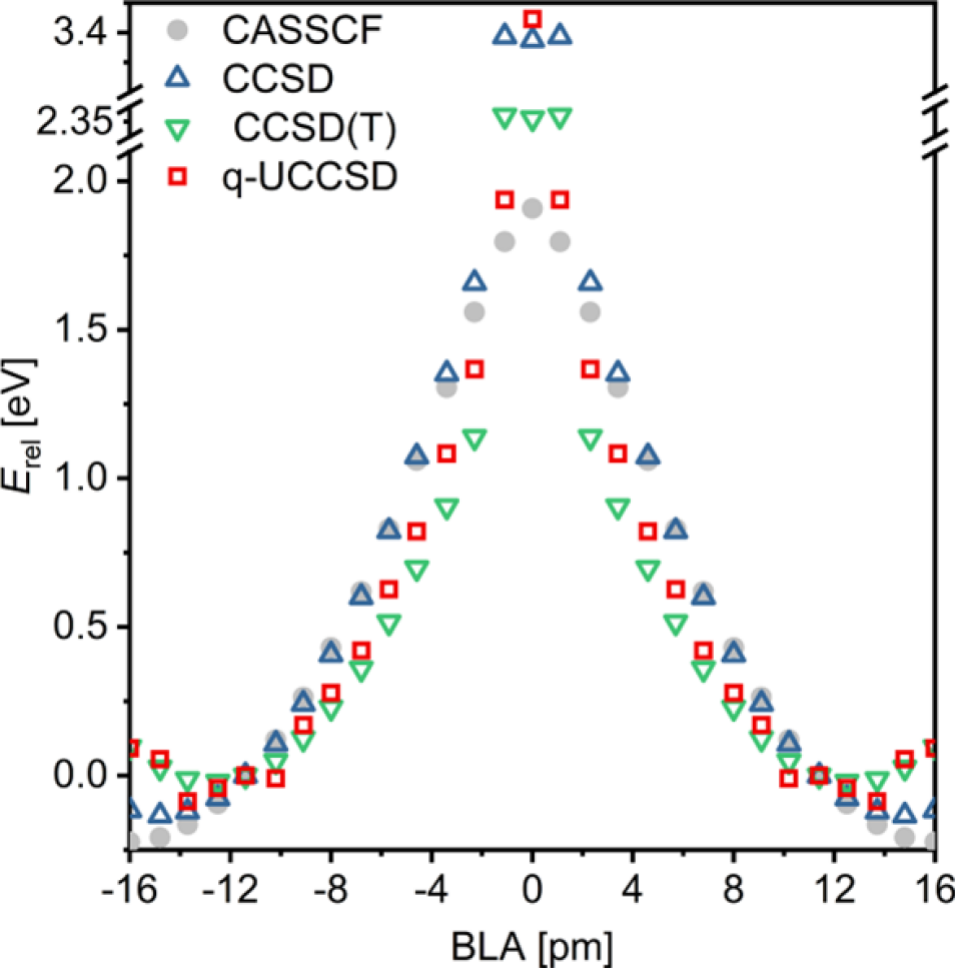
Energies of the S0 state C_16_ at different amounts of BLA calculated using CASSCF (gray circles), CCSD (blue hollow triangles), CCSD(T) (green hollow triangles), and divide-and-conquer q-UCCSD (red rectangles), all obtained with the cc-pVDZ basis set. Note the two breaks on the *y* axis.

In the S_0_ state, this divide-and-conquer q-UCCSD approach works well for a wide range of nonzero BLA (Figure 9, MAE = 0.16 eV relative to CASSCF), producing results very similar to those of canonical CCSD and CCSD(T) methods. Around zero BLA, both q-UCCSD and canonical coupled clusters are limited by the poor quality of the underlying HF determinant, although q-UCCSD appears to be more robust in case of BLA ≈ 1 pm. In these cases, the performance of q-UCCSD may be further improved by starting from a better reference (e.g., CASSCF) or by using a flavor which includes orbital optimization.^59^ q-UCCSD calculations for the T_1_ and Q_1_ states are of similar quality to S_0_ (see the SI), illustrating a way for applying the q-UCCSD method to systems with many strongly correlated electrons (other possible use cases may be a complex with two weakly coupled transition metals, or a dye with two chromophores connected with sigma bonds).

## 3 Conclusions

Using CASSCF, qualitative modeling, and DFT, we have demonstrated that the ring current in C_16_ can be reversed by small changes in BLA. This reversal of aromaticity (according to the ring-current criterion^2^) is unique as it is not associated by any notable change in the electronic structure, but only with a small change in energy. It occurs in electronic states displaying mixed aromaticity, which can be characterized by the presence of both diatropic and paratropic currents (Table 1).

The Hückel–Heilbronner tight-binding model captures the essence of the electronic structure of C_16_, qualitatively reproducing the variation of energy obtained by much more complex methods (CASSCF and range-separated DFT). Magnetic couplings between the C_16_ orbitals are successfully described by using a minimal ipsocentric model. This model provides a direct link between aromaticity and orbital energy, thus offering a simple avenue for rationalizing commonly observed correlations between aromaticity and various molecular properties.^8,9,10,11^

Aromaticity reversals may also be interpreted in terms of antiaromaticity relief.^30,60^ The doubly aromatic Q_1_ state adopts a BLA = 0 geometry, which maximizes its aromaticity, while the doubly antiaromatic S_0_ minimizes its antiaromaticity by increasing BLA as much as the σ system allows. The mixed aromatic T_1_ and S_1_ states have approximately nonaromatic *D*_8h_ minima, revealing their aromatic and antiaromatic nature by movement along one and the other directions of the Kekulé vibration.

In any system with more than one π pathway, the ring currents produced by individual circuits may be opposed, leading to a possibility of aromaticity reversals of the type discussed here. More generally, we can expect the behavior of such “mixed aromatic” systems to be very sensitive to changes in geometry.

We also demonstrate that the applicability of quantum algorithms such as q-UCCSD can be improved by partitioning the active space and calculating the correlation energy of each subspace separately. In the case of C_16_, this divide-and-conquer approach is particularly successful, approaching the accuracy of fully self-consistent multireference calculations. Finally, as quantum devices have direct access to the wave function, it is straightforward to compute the expectation value of any observable. This suggests that the ipsocentric approach could be combined with virtually any quantum ansatz.

**Table 1:** Ring Current of C_16_ in Low-Living Electronic States at Different Amounts of Bond-Length Alternation

		**net ring current**	
**state**	**electronic structure**	**BLA ≫ 0**	**BL**A ≈ 0
S_0_	doubly antiaromatic	paratropic	paratropic
Q_1_	doubly Baird aromatic	diatropic	diatropic
T_1_	Baird aromatic + antiaromatic	diatropic	paratropic
S_1_	doubly mixed	diatropic	paratropic

## Supporting Information Available

Molecular geometries, DFT results for the S_0_, T_1_, and Q_1_ states, and MATLAB code used for the HHM (PDF)
