# Supplementary material for: Aromaticity Reversal Induced by Vibrations in Cyclo[16]carbon
Source: J Am Chem Soc. 2023 Dec 1;145(49):26962–72. doi: 10.1021/jacs.3c10207 (PMC10722511; doi:10.1021/jacs.3c10207)
Supplement: Supplementary file 1 — ja3c10207_si_001.pdf [file ja3c10207_si_001.pdf]

Supporting Information for Aromaticity Reversal Induced by Vibrations in Cyclo[16]carbon

Igor Rončević,[a,*] Freddie J. Leslie,[a,‡] Max Rossmannek,[b,‡] Ivano Tavernelli,[b] Leo Gross,[b] and Harry L. Anderson[a]

[a] Department of Chemistry, Oxford University, Chemistry Research Laboratory, Oxford OX1 3TA, United Kingdom.

[b] IBM Research Europe – Zurich, Säumerstrasse 4, 8803 Rüschlikon, Switzerland.

‡ These authors contributed equally.

COMPUTATIONAL DETAILS........S2

ADDITIONAL DETAILS OF QUANTUM CALCULATIONS........S3

SUPPLEMENTARY FIGURES........S4

MINIMAL IPSOCENTRIC MODEL........S5

HHM FITTING DETAILS........S7

MATLAB SCRIPT........S8

GEOMETRIES........S18

REFERENCES........S28

APPENDIX........S29

COMPUTATIONAL DETAILS

Geometries were obtained by interpolating equidistant points between the NEVPT2(12,12)/def2-TZVP D_{8h} minimum determined in previous work[1] and a D_{16h} (BLA = 0) minimum found by line minimization (with ring radius r as the only variable). The two geometries have nearly equally sized rings (3.33 Å–3.32 Å), indicating that these two points are connected almost exactly by a Kekulé vibration modulating the amount of BLA. QD-NEVPT2(12,12)/def2-TZVP calculations included five singlet roots, three triplet, and a single quintet root. These calculations were done using ORCA.[2]

DFT calculations (ωB97XD/def2-TZVP; Figure S1) were done using Gaussian16.[3] Specific determinants were investigated using orbital rotations (i.e. ΔSCF). CASSCF energies and ring currents were obtained at the CASSCF(12,12)/cc-pVDZ level using DALTON.[4] Ring currents were determined from NICS(2)$_{zz}$ values, with a correlation to ring currents obtained using SYSMOIC[5] given in Figure S2a. NICS(0)zz and NICS(1)zz values give worse correlation (Figure S2b,c), likely due to the effect of the physically proximal in-plane and out-of-plane orbitals, respectively. We also note that SYSMOIC reproduces the NICS results even for T_1 and Q_1 electronic states, despite not being designed to treat open-shell systems.

Initial HF calculations (restricted spin for the singlet state, restricted open-shell for other states) were run using PySCF[6] with the cc-pVDZ basis set. Post-HF reference calculations, including coupled clusters singles doubles (CCSD) and coupled clusters singles doubles and perturbative triples (CCSD(T)) were also obtained with PySCF.

The quantum embedding calculations were performed using Qiskit.[7] Specifically, the CASCI module in PYSCF was used, with the FCI solver replaced by a variational quantum eigensolver (VQE)[8] in combination with a quantum unitary coupled cluster singles and doubles (qUCCSD) ansatz.[9] The SPSA optimizer was employed for the variational optimization of the wavefunction parameters.[10]

Two variants of the q-UCCSD ansatz were tested: the standard implementation, which does not include excitations between orbitals of different spin (labelled “no spin flips” in Figure S4), and an extended implementation which does allow such excitations (referred to as “q-UCCSD” throughout the text and graphics).

All quantum embedding calculations were simulated on classical hardware by means of exact state vector computations.[7] Thus, no statistical sampling errors or inherent quantum errors are incorporated into the results.

ADDITIONAL DETAILS OF QUANTUM CALCULATIONS

S_0 state. The divide-and-conquer q-UCCSD approach reproduces the reference CASSCF results reasonably well (Figure S1a) at nonzero BLA. The deviation at BLA = 0 likely stems from a poor starting point, as a single-reference (HF) wavefunction is unable to describe a degenerate ground state (two electrons in four degenerate spin-orbitals for both π systems) well. This degeneracy can also cause convergence issues for the VQE optimizer.

T_1 state. In case of the triplet, the CCSD(T) and CASSCF results roughly coincide, with CCSD showing considerably worse results (Figure S1b). Including spin-flips in the divide-and-conquer q-UCCSD approach appears to be essential, leading to an improvement of around 1.2 eV in the vertical excitation energy relative to CASSCF. As the HF reference for the triplet is |11 20> (out-of-plane triplet, in-plane singlet), a possible explanation for the superior performance of the spin flip-allowed flavor of q-UCCSD is that it can access the |20 11> configuration (out-of-plane singlet, in-plane triplet), which has an important contribution to the overall wavefunction (cf. Figure 4a in main text). It is particularly notable that the q-UCCSD approach with spin flips finds the same minimum as CASSCF and produces overall better results than CCSD. The zero BLA geometry is still difficult due to a poor starting point, although this electronic state is less pathological than S0 (two electrons in four near-degenerate spin-orbitals for one π system).

Q_1 state. In a similar vein to T_1, CCSD(T) reproduces the CASSCF results correctly (Figure S1b). Our divide-and-conquer is again superior to CCSD, but it performs slightly worse than CCSD(T), producing curves with slightly too much curvature. This could arise from a limitation of the divide-and-conquer approach in which both systems are effectively treated as individual triplets and later recombined, as this does not guarantee that the recombined system will purely be a quintet. Furthermore, we again observe a limitation of q-UCCSD not being able to recover from a bad starting point at small BLA = 0 value. This effect increases significantly when spin-flipping excitations are not allowed, illustrating the importance of using the q-UCCSD flavor which does allow such excitations.

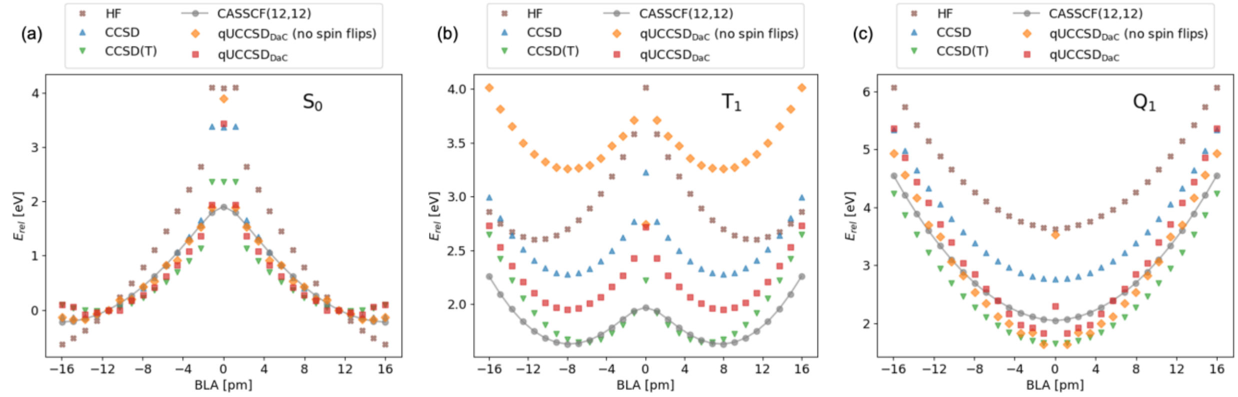

Figure S1. Energies of C_{16} in S_0 (a), Q_1 (b), and T1 (c) states at different BLA values calculated using HF (brown crosses), CCSD (blue upward-pointing triangles), CCSD(T) (green downward-pointing triangles), divide-and-conquer q-UCCSD without spin flips (orange diamonds) and with spin flips (red squares). The cc-PVDZ basis set was used in all cases. All energies are shown relative to the S0 energy at BLA = 11.4 pm.

SUPPLEMENTARY FIGURES

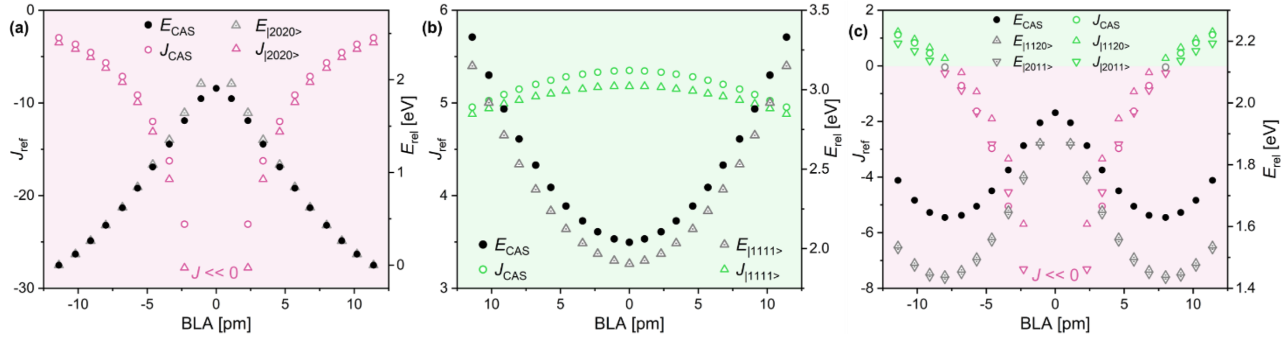

Figure S2. Energies and ring currents in S_0 (a), Q_1 (b), and T_1 (c) states of C_{16}, calculated by CASSCF (circles) and DFT (triangles) at different levels of BLA. In case of T_1, two DFT configurations were investigated, corresponding to electron flips in the out-of-plane (|11 20>) or the in-plane (|20 11>) π system. Energies (E) are shown relative to the S0 geometry with BLA = 11.2 pm, and ring currents (J) relative to the benzene ring current (J_{ref} = 12.00 nA/T).

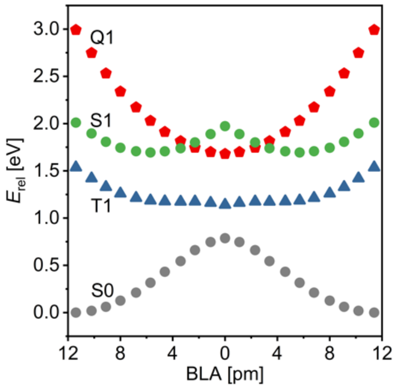

Figure S3. QD-NEVPT2/def2-TZVP energies of the S_0 (gray circles), T_1 (blue triangles), S_1 (green circles), and Q_1 (red pentagons) electronic states at different BLA values.

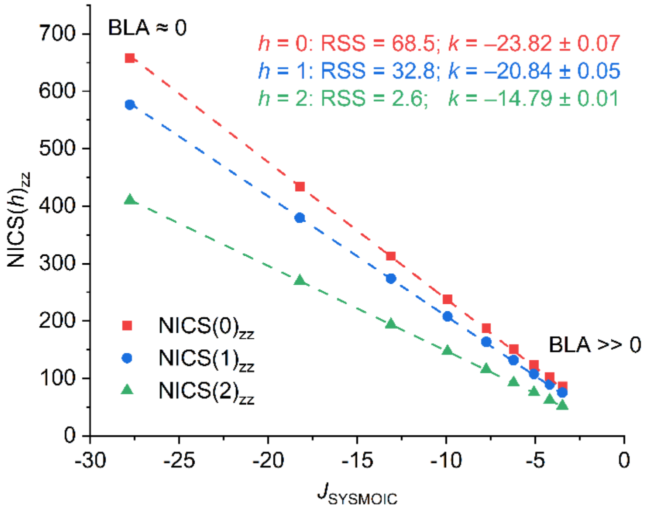

Figure S4. Linear fits between S0 ring currents obtained using $NICS(h)_{zz}$ and SYSMOIC, with h = 0, 1 or 2 Å. Intercept is set to zero in all cases, k is the slope, and RSS is the residual sum of squares. Throughout this work, $NICS(2)_{zz}$ values were used to calculate the ring current.

MINIMAL IPSOCENTRIC MODEL

In the ipsocentric approach, the current density *j* at point *r* (which acts as its own gauge origin) induced by an occupied orbital ψ_s in presence of a magnetic field *B* is given by:[11]

$$j_s(r) = \frac{ie\hbar}{m_e}\int \psi_s(r)\nabla\psi_s^{(1)}(r) - \psi_s^{(1)}(r)\nabla\psi_s(r)d\tau \qquad \text{(S1)}$$

where $\psi_s^{(1)}$ is the first-order perturbed orbital. The contribution of every unoccupied orbital ψ_t to $\psi_s^{(1)}$ can be written as:

$$\psi_{s\to t}^{(1)} = \psi_{s\to t}^{(\mathrm{d})} + \psi_{s\to t}^{(\mathrm{p})} = \frac{e}{2m_e}\left[\left(d\times\psi_t\frac{M_\mathrm{T}}{\Delta\varepsilon}\cdot B\right) - \left(\psi_t\frac{M_\mathrm{R}}{\Delta\varepsilon}\cdot B\right)\right] \qquad \text{(S2)}$$

Where *B* is the applied magnetic field, $\Delta\varepsilon$ is the difference in orbital energies between ψ_t and ψ_s, *d* is a displacement from the origin of the coordinate system, and M_T and M_R are matrix elements for translational and rotational transitions:

$$M_T = \langle\psi_t|P|\psi_s\rangle$$
$$M_R = \langle\psi_t|L(0)|\psi_s\rangle \qquad \text{(S3)}$$

In (3), *P* is the linear momentum operator, while *L*(0) is the angular momentum operator representing rotation about the origin of the coordinate system. Let us now consider a ring of *N* equally spaced atoms carrying orbitals f_n. In the Hückel model, solutions for this system can be written as degenerate sine (A) or cosine (B) functions with angular momentum *k*:

$$\psi_{k,A} = \sum_n \phi_n \sin\left(\frac{2\pi kn}{N}\right)$$
$$\psi_{k,B} = \sum_n \phi_n \cos\left(\frac{2\pi kn}{N}\right) \qquad \text{(S4)}$$

Where *n* is the index of the *n*th atom. If the ring is positioned in the xy plane and centred in the origin of the coordinate system, and the magnetic field is applied in the z direction, the ring current induced per unit of magnetic field at unit displacement in the xy plane can be written as:

$$\frac{j_s}{|B|} = -\frac{ie^2\hbar}{2m_e^2}\frac{M_\mathrm{T}+M_\mathrm{R}}{\Delta\varepsilon}\int\psi_s\nabla\psi_t - \psi_t\nabla\psi_s d\tau \qquad \text{(S5)}$$

To obtain (S5), we have assumed that orbitals are spherically symmetric, which does not qualitatively compromise the quality of the obtained ring currents.[12] Therefore, in a simple monocyclic system the diatropic current induced by an occupied-unoccupied orbital pair will be linear in $M_\mathrm{T}/\Delta e$, and the paratropic current will be linear in $M_\mathrm{R}/\Delta e$, which results in the model:

$$j_\mathrm{model} = \sum_i \frac{c_{dia}}{\Delta\varepsilon_i} - \sum_i \frac{c_{para}}{\Delta\varepsilon_i} \qquad \text{(S6)}$$

where *i* runs over all allowed transitions.

MINIMAL IPSOCENTRIC MODEL APPLIED TO BENZENE

In the main text of the paper, we use ring currents obtained by CASSCF and orbital energies obtained by HHM as input for the minimal ipsocentric model. However, both of these quantities are also accessible by DFT. For example, in case of ground-state (S_0) benzene, we get the following orbital energies (in eV, obtained at ωB97X-D/def2-TZVP, numbers shown in red correspond to unoccupied orbitals):

S_0	Aα	Aβ	Bα	Bβ
$k = 2$	1.58	1.58	1.58	1.58
$k = 1$	-9.06	-9.06	-9.06	-9.06

As only diatropic transitions are present (Figure 1a left in main text) and $J_{S0} = J_{rel}$ = 12.00 nA/T, we obtain c^{DIA} = 5.32 J_{rel}/eV. In the T_1 state equilibrium geometry, both diatropic and paratropic transitions are present (Figure 1a right in main text) and the orbital energies are:

T_1	Aα	Aβ	Bα	Bβ
$k = 2$	-5.12	2.17	1.77	2.27
$k = 1$	-9.91	-9.21	-9.83	-2.30

Using the above-determined value of c^{DIA} = 5.32 J_{rel}/eV, and calculating J_{T1} = –1.09 J_{rel} from a NICS calculation, we can now obtain c^{PARA} = 11.26 J_{rel}/eV, which completes the parametrisation of the minimal ipsocentric model for benzene. We can now apply c^{DIA} and c^{PARA} to benzene in the S_1 state. Orbital energies (at the T_1 geometry) are accessible to DFT via an orbital rotation from the S_0 state are:

S_1	Aα	Aβ	Bα	Bβ
$k = 2$	-3.98	0.89	2.10	1.70
$k = 1$	-9.77	-9.38	-3.62	-8.72

Here we obtain J_{S1} = –1.33 J_{rel}, while a NICS calculation done using DFT produces a nonsensical value. A CASSCF calculation found in literature[13] predicts J_{S1} = 1.13 J_{T1}, indicating that the Baird's rule holds for the S_0 state, which is in very good agreement with the relation obtained using the minimal ipsocentric model (J_{S1} = 1.22 J_{T1}).

HHM FITTING DETAILS

The HHM model described by an *n* x *n* matrix given in equation (4) can be solved to give energies equal to:

$$E_k = \alpha \pm \beta\sqrt{2}\sqrt{1 + \delta^2 + (1 - \delta^2)\cos\left(\frac{4\pi k}{n}\right)} \qquad \text{(S1)}$$

Where k takes integer values between 0 and $\frac{n}{2} - 1$ (inclusive). A single matrix can be used to describe the π-orbitals of an annulene, and two matrices can be used to describe in-plane and out-of-plane π-orbitals for a cyclocarbon. All energies were listed and sorted. To calculate the S0 state energy, the lowest 16 orbitals were summed at each value of δ. For the S1, T1, and Q1 states, orbitals were selected manually. The π energies were added to a parabola in δ, which describes perturbation to the sigma bonds as a simple harmonic oscillator, and the lowest point of the energy curve for S0 was taken as the 0 point.

These energies were fitted to the CASSCF data by varying A (the coefficient of the sigma parabola) β', β'', α', and exchange correlation values (offsets) for S1, T1, and Q1. This was performed using Microsoft Excel's GRG non-linear algorithm with multistart. α'' was set to 0 such that α' = γ, as a change in α'' at constant γ has no effect on any relative energies, and therefore has no effect on the magnetic properties.

The values of the parameters that gave the best energy fit were:

A =	306.39 eV
α' =	0.22 eV
β'' =	6.10 eV
β' =	4.40 eV
Q1 offset =	-3.28 eV
T1 offset =	-0.26 eV
S1 offset =	0.40 eV

As $E_\sigma = A\delta^2$, where $\delta = \frac{BLA(pm)}{129.5}$, this can be rearranged to give $E_\sigma = \frac{1}{2}k * BLA^2$, where k is the bond's force constant, equal to $\frac{2A}{129.5^2}$. At *A* = 306.39 eV, this corresponds to a wavenumber of 1016 cm^{-1}, which is in the typical range for a carbon-carbon single bond.

The HHM fitted energies were then used to calculate the overall ring current using the minimal ipsocentric model. This was performed by manual selecting the relevant transitions and appropriately assigning them as paramagnetic or diamagnetic. The c_{para} and c_{dia} values for the out of plane system were allowed to vary freely, and the values for the in plane system were given by multiplying the relevant values by a factor, *c'*. The model was fitted to the $NICS_{zz}(2)$ ring current data using Microsoft Excel's GRG non-linear algorithm with multistart.

The values of the parameters that gave the best magnetic current density were:

c_{para} =	−8.05
c_{dia} =	6.05
c' =	0.555

MATLAB SCRIPT

The following script was used to calculate the orbital energies of the HHM model for cyclo[16]carbon numerically. It also plots the total energy for various the S0, S1, T1, and Q1 states as well as generating some crude orbital plots for the frontier molecular orbitals of the in-plane π-system.

```
% A script to generate a modified huckel theory calculation that attempts

% to account for energy changes due to bond length and angle distortions
% within a cyclocarbon molecule

clear
n=16; % Define number of carbon atoms in the cyclocarbon (MUST BE EVEN)
a1=0; % Define out of plane on-site matrix element value
a2=0.290708; % Define in plane on-site matrix element value
b1=6.622913; % Define out of plane off-site matrix element value
b2=5.098994; % Define on plane off-site matrix element value
A=363.81095; % Define sigma parabola energy coefficient value
dmax=0.087895; %Define maximum value of delta
S1xch=0; %Define offset parameter for S1
T1xch=0; %Define offset parameter for T1
Q1xch=0; %Define offset parameter for Q1
res=10; % Choose resolution of calculations
Print_orbs(1,1)='Y'; % Show Orbital plots (Y/N)
Print_Frosts(1,1)='Y';% Show Frost Diagrams (Y/N)
showcircs(1,1)='Y'; % Show circles on Frost diagrams (Y/N)
pltmagints(1,1)='Y';% Show Magnetic dipole integrals (Y/N)
OutPMatrix=zeros(n,n);
InPMatrix=zeros(n,n);

for i = 1:n-1 % definining initial unmodified matrix
    OutPMatrix(i,i)=a1;
    InPMatrix(i,i)=a2;
    OutPMatrix(n,n)=a1;
    InPMatrix(n,n)=a2;
    OutPMatrix(n,1)=b1;
    InPMatrix(n,1)=b2;
    OutPMatrix(1,n)=b1;
    InPMatrix(1,n)=b2;
    OutPMatrix(i,i+1)=b1;
    OutPMatrix(i+1,i)=b1;
    InPMatrix(i,i+1)=b2;
    InPMatrix(i+1,i)=b2;
end

outhomoengs=zeros(res+1,1);
outsubhomoengs=zeros(res+1,1);
outlumoengs=zeros(res+1,1);
outsuplumoengs=zeros(res+1,1);
inhomoengs=zeros(res+1,1);
insubhomoengs=zeros(res+1,1);
inlumoengs=zeros(res+1,1);
insuplumoengs=zeros(res+1,1);
xcoord=zeros(res+1,1);
xcoord2=zeros(res+1,1);
xcoordgen=zeros(res+1,1);
```

```
OutOrbHomos=zeros(n,res+1);
InOrbHomos=zeros(n,res+1);
OutOrbLumos=zeros(n,res+1);
InOrbLumos=zeros(n,res+1);
allevals=zeros(2*n,res+1);
EpitotalS0=zeros(res+1,1);
EpitotalS1=zeros(res+1,1);
EpitotalT1=zeros(res+1,1);
EpitotalQ1=zeros(res+1,1);
EsigtotalS0=zeros(res+1,1);
EsigtotalS1=zeros(res+1,1);
EsigtotalT1=zeros(res+1,1);
EsigtotalQ1=zeros(res+1,1);
EsigtotalS0rel=zeros(res+1,1);
EsigtotalS1rel=zeros(res+1,1);
EsigtotalT1rel=zeros(res+1,1);
EsigtotalQ1rel=zeros(res+1,1);
SigEns=zeros(res+1,1);
outedifftest=zeros(res+1,1);
inedifftest=zeros(res+1,1);
outallens=zeros(res+1,n);
inallens=zeros(res+1,n);
outfrostx=zeros(res+1,n);
infrostx=zeros(res+1,n);
outallorbs=zeros(n+1,n,res+1);
inallorbs=zeros(n+1,n,res+1);
ntestvec=zeros(n,1);
outparacont=zeros(res+1,1);
outdiacont=zeros(res+1,1);
inparacont=zeros(res+1,1);
indiacont=zeros(res+1,1);
outtotcont=zeros(res+1,1);
intotcont=zeros(res+1,1);
outtripdcont=zeros(res+1,1);
outsingpcont=zeros(res+1,1);
outsingdcont=zeros(res+1,1);
insingpcont=zeros(res+1,1);
Orbcoords=zeros(n,2,res+1);
outhomoorbs=zeros(n,3,res+1);
outhomodownorbs=zeros(n,3,res+1);
outlumoorbs=zeros(n,3,res+1);
outlumouporbs=zeros(n,3,res+1);
inhomoorbs=zeros(n,3,res+1);
inhomodownorbs=zeros(n,3,res+1);
inlumoorbs=zeros(n,3,res+1);
inlumouporbs=zeros(n,3,res+1);

g=-dmax/res; % Steps of delta

disp('Calculating out of plane orbitals')
for g1a = 0:res
    for i = 1:n-1 %editing Out of plane matrix to account for g1
        OutPMatrix(1,n)=b1*(1+g*g1a);
        OutPMatrix(n,1)=b1*(1+g*g1a);
        if mod(i,2)==0
```

```
            OutPMatrix(i,i+1)=b1*(1+g*g1a);
            OutPMatrix(i+1,i)=b1*(1+g*g1a);
        else
            OutPMatrix(i,i+1)=b1*(1-g*g1a);
            OutPMatrix(i+1,i)=b1*(1-g*g1a);
        end
    end
    [Outvects,Outvals]=eig(OutPMatrix,'nobalance');
    Outens=zeros(1,n);
    [d,ind]=sort(diag(Outvals));
    Outvals=Outvals(ind,ind);
    Outvects=Outvects(:,ind);
    for j = 1:n
        Outens(1,j)=real(Outvals(j,j)); %Finding eigenvalues of g1
        allevals(j,g1a+1)=Outvals(j,j); % Producing a matrix containing all energy
values over orbitals and BLA variations
    end
    outhomoengs(g1a+1,1)=Outens(n/2); %Out of plane HOMO energies as g1 changes
    outsubhomoengs(g1a+1,1)=Outens(n/2-1); %Out of plane HOMO -1 energies as g1
changes
    outlumoengs(g1a+1,1)=Outens(n/2+1); %Out of plane LUMO energies as g1 changes
    outsuplumoengs(g1a+1,1)=Outens(n/2+2); %Out of plane LUMO+1 energies as g1
changes
    xcoord(g1a+1,1)=-g1a*g; % Define x coordinate
    xcoordgen(g1a+1,1)=g1a;
    outallens(g1a+1,:)=Outens; %Matrix of all out of plane energy values

    % Code for sorting eigenvectors by energy, then into arrays of homos and lumos
    normoutsqh = 0;
    normoutsql = 0;
    for i = 1:n
        normoutsqh = normoutsqh + Outvects(i,n/2)^2;
        normoutsql = normoutsql + Outvects(i,n/2+1)^2;
    end
    ntestvec(g1a+1) = normoutsqh;
    normouth = sqrt(normoutsqh);
    normoutl = sqrt(normoutsql);
    for k = 1:n
        OutOrbHomos(k,g1a+1)=Outvects(k,n/2)/normouth;
        OutOrbLumos(k,g1a+1)=Outvects(k,n/2+1)/normoutl;

    end
    for k=1:n
        outallorbs(1,k,g1a+1)=Outens(k);
        for j=1:n
            outallorbs(j+1,k,g1a+1)=Outvects(j,k);
        end
    end
end

disp('Calculating in plane orbitals')
for g2a = 0:res % repeating previous process for in plane orbitals matrix
    for i = 1:n-1
        InPMatrix(1,n)=b2*(1+g*g2a); %Varying b2 such that at maximum bond
alternation,
```

```
        InPMatrix(n,1)=b2*(1+g*g2a); %Values are equal to those in the out of plane
matrix
        if mod(i,2)==0
            InPMatrix(i,i+1)=b2*(1+g*g2a);
            InPMatrix(i+1,i)=b2*(1+g*g2a);
        else
            InPMatrix(i,i+1)=b2*(1-g*g2a);
            InPMatrix(i+1,i)=b2*(1-g*g2a);
        end
    end
    [Invects, Invals]=eig(InPMatrix,'nobalance');
    Inens=zeros(1,n);
        [d,ind]=sort(diag(Invals));
    Invals=Invals(ind,ind);
    Invects=Invects(:,ind);
    for j = 1:n
        Inens(1,j)=real(Invals(j,j));
        allevals(n+j,g2a+1)=Invals(j,j);
    end
    inhomoengs(g2a+1,1)=Inens(n/2);
    insubhomoengs(g2a+1,1)=Inens(n/2-1);
    inlumoengs(g2a+1,1)=Inens(n/2+1);
    insuplumoengs(g2a+1,1)=Inens(n/2+2);
    xcoord2(g2a+1,1)=-g2a*g;
    inallens(g2a+1,:)=Inens; %Matrix of all out of plane energy values

 % Code for sorting eigenvectors by energy, then into arrays of homos and lumos
    norminsqh = 0;
    norminsql = 0;
    for i = 1:n
        norminsqh = norminsqh + Invects(i,n/2)^2;
        norminsql = norminsql + Invects(i,n/2+1)^2;
    end
    norminh = sqrt(norminsqh);
    norminl = sqrt(norminsql);
    for k = 1:n
        InOrbHomos(k,g2a+1)=Invects(k,n/2)/norminh;
        InOrbLumos(k,g2a+1)=Invects(k,n/2+1)/norminl;
    end
    for k=1:n
        inallorbs(1,k,g2a+1)=Inens(k);
        for j=1:n
            inallorbs(j+1,k,g2a+1)=Invects(j,k);
        end
    end
end

sortallevals=sort(allevals);

for r=1:res+1
    for i=1:n
        EpitotalS0(r)= EpitotalS0(r) + 2*sortallevals(i,r); %Total energy for S0, Sum
of lowest 16 energies times 2
    end
    %Total pi orbital energies
```

```
    EpitotalS1(r) = EpitotalS0(r) - sortallevals(n,r) + sortallevals(n+1,r); %N.B.
orbital occupancy recalculated at each geometry
    EpitotalT1(r) = EpitotalS0(r) - sortallevals(n,r) + sortallevals(n+1,r);
    EpitotalQ1(r) = EpitotalS0(r) - sortallevals(n,r) - sortallevals(n-1,r) +
sortallevals(n+1,r) + sortallevals(n+2,r);
    %Pi orbital energies with additional parabola to account for sigma energies
    EsigtotalS0(r)= EpitotalS0(r) + A*(g*(r-1))^2;
    EsigtotalS1(r)= EpitotalS1(r) + A*(g*(r-1))^2 + S1xch;
    EsigtotalT1(r)= EpitotalT1(r) + A*(g*(r-1))^2 + T1xch;
    EsigtotalQ1(r)= EpitotalQ1(r) + A*(g*(r-1))^2 + Q1xch;
    SigEns(r)= A*(g*(r-1))^2;
end

for r=1:res+1
    EsigtotalS0rel(r)=EsigtotalS0(r)-EsigtotalS0(res+1);
    EsigtotalS1rel(r)=EsigtotalS1(r)-EsigtotalS0(res+1);
    EsigtotalT1rel(r)=EsigtotalT1(r)-EsigtotalS0(res+1);
    EsigtotalQ1rel(r)=EsigtotalQ1(r)-EsigtotalS0(res+1);
end

close all

hold on
disp('Printing Outputs')

figure(1);
tiledlayout(1,3)

nexttile
plot(xcoord,outhomoengs,xcoord,outlumoengs,xcoord,outsubhomoengs,xcoord,outsuplumoeng
s) %plot out of plane graph with variance in g1
legend('Outer HOMO', 'Outer LUMO', 'Outer HOMO-1', 'Outer LUMO+1')
title('Out of Plane orbitals')

nexttile
plot(xcoord,inhomoengs,xcoord,inlumoengs,xcoord,insubhomoengs,xcoord,insuplumoengs)
%plot in plane graph with variance in g2
legend('Inner HOMO', 'Inner LUMO', 'Inner HOMO-1', 'Inner LUMO+1')
title('In Plane Orbitals')

nexttile %plotting all orbitals
plot(xcoord,outhomoengs,xcoord,outlumoengs,xcoord,outsubhomoengs,xcoord,outsuplumoeng
s,xcoord,inhomoengs,xcoord,inlumoengs,xcoord,insubhomoengs,xcoord,insuplumoengs)
legend('Outer HOMO', 'Outer LUMO', 'Outer HOMO-1', 'Outer LUMO+1', 'Inner HOMO',
'Inner LUMO', 'Inner HOMO-1', 'Inner LUMO+1')
title('All Orbitals')

hold off
disp('Printed frontier orbital energy variations')

engtable=table(xcoord,outallens,inallens,EpitotalS0);%Producing table of energy data
writetable(engtable,'HuckelAllEnergies.csv','Delimiter',',')

hold on
```

```
if Print_orbs(1,1)=='N'
        disp('Frontier orbital wavefunctions printing off')
elseif res>10
    disp('Too many iterations to print Frontier orbitals - lower res')
else
    disp('Printing frontier orbital wavefunctions')
figure(2);
tiledlayout(4,res+1) % plotting orbitals of Outer HOMOS and LUMOS +-1
for i = 1:res+1
    nexttile
    bar(outallorbs(2:n+1,n/2-1,i))
end
for i = 1:res+1
    nexttile
    bar(outallorbs(2:n+1,n/2,i))
end
for i = 1:res+1
    nexttile
    bar(outallorbs(2:n+1,n/2+1,i))
end
for i = 1:res+1
    nexttile
    bar(outallorbs(2:n+1,n/2+2,i))
end
figure(3);
tiledlayout(4,res+1) % plotting orbitals of Inner HOMOS and LUMOS +-1
for i = 1:res+1
    nexttile
    bar(inallorbs(2:n+1,n/2-1,i))
end
for i = 1:res+1
    nexttile
    bar(inallorbs(2:n+1,n/2,i))
end
for i = 1:res+1
    nexttile
    bar(inallorbs(2:n+1,n/2+1,i))
end
for i = 1:res+1
    nexttile
    bar(inallorbs(2:n+1,n/2+2,i))
end

for i=1:n/2
    for k=1:res+1

Orbcoords(2*i,1,k)=cos(2*pi*2*i/16 - 0.75*(k-1)*g);
Orbcoords(2*i-1,1,k)=cos(2*pi*(2*i-1)/16 + 0.75*(k-1)*g);
Orbcoords(2*i,2,k)=sin(2*pi*2*i/16 - 0.75*(k-1)*g);
Orbcoords(2*i-1,2,k)=sin(2*pi*(2*i-1)/16 + 0.75*(k-1)*g);
    end
end

if res+1>5
else
```

```
    for k=1:res+1
outhomoorbs(:,1,k)=Orbcoords(:,1,k);
outhomodownorbs(:,1,k)=Orbcoords(:,1,k);
outlumoorbs(:,1,k)=Orbcoords(:,1,k);
outlumouporbs(:,1,k)=Orbcoords(:,1,k);
inhomoorbs(:,1,k)=Orbcoords(:,1,k);
inhomodownorbs(:,1,k)=Orbcoords(:,1,k);
inlumoorbs(:,1,k)=Orbcoords(:,1,k);
inlumouporbs(:,1,k)=Orbcoords(:,1,k);

outhomoorbs(:,2,k)=Orbcoords(:,2,k);
outhomodownorbs(:,2,k)=Orbcoords(:,2,k);
outlumoorbs(:,2,k)=Orbcoords(:,2,k);
outlumouporbs(:,2,k)=Orbcoords(:,2,k);
inhomoorbs(:,2,k)=Orbcoords(:,2,k);
inhomodownorbs(:,2,k)=Orbcoords(:,2,k);
inlumoorbs(:,2,k)=Orbcoords(:,2,k);
inlumouporbs(:,2,k)=Orbcoords(:,2,k);

outhomoorbs(:,3,k)=outallorbs(2:n+1,n/2,k);
outhomodownorbs(:,3,k)=outallorbs(2:n+1,n/2-1,k);
outlumoorbs(:,3,k)=outallorbs(2:n+1,n/2+1,k);
outlumouporbs(:,3,k)=outallorbs(2:n+1,n/2+2,k);
inhomoorbs(:,3,k)=inallorbs(2:n+1,n/2,k);
inhomodownorbs(:,3,k)=inallorbs(2:n+1,n/2-1,k);
inlumoorbs(:,3,k)=inallorbs(2:n+1,n/2+1,k);
inlumouporbs(:,3,k)=inallorbs(2:n+1,n/2+2,k);
    end

    figure(4)
    tiledlayout(4,res+1)
    colormap spring
    for k = 1:res+1
        nexttile

scatter(outhomodownorbs(:,1,k),outhomodownorbs(:,2,k),100,outhomodownorbs(:,3,k),'fil
led')
        axis equal
    end
    for k = 1:res+1
        nexttile

scatter(outhomoorbs(:,1,k),outhomoorbs(:,2,k),100,outhomoorbs(:,3,k),'filled')
        axis equal
    end
    for k = 1:res+1
        nexttile

scatter(outlumoorbs(:,1,k),outlumoorbs(:,2,k),100,outlumoorbs(:,3,k),'filled')
        axis equal
    end
    for k = 1:res+1
        nexttile

scatter(outlumouporbs(:,1,k),outlumouporbs(:,2,k),100,outlumouporbs(:,3,k),'filled')
```

```
        axis equal
    end
       cb=colorbar();

    writematrix(outhomoorbs,'OutHOMOorbs.csv','Delimiter',',');
    writematrix(outhomodownorbs,'OutHOMOdownorbs.csv','Delimiter',',');
    writematrix(outlumoorbs,'OutLUMOorbs.csv','Delimiter',',');
    writematrix(outlumouporbs,'OutLUMOuporbs.csv','Delimiter',',');
    writematrix(inhomoorbs,'inHOMOorbs.csv','Delimiter',',');
    writematrix(inhomodownorbs,'inHOMOdownorbs.csv','Delimiter',',');
    writematrix(inlumoorbs,'inLUMOorbs.csv','Delimiter',',');
    writematrix(inlumouporbs,'inLUMOuporbs.csv','Delimiter',',');


end
end

%Total energy plots
figure(5)
tiledlayout(1,5)
nexttile
plot(xcoord,EsigtotalS0,xcoord,EpitotalS0)
legend('Total S0 energy','Pi Energies')
title('S0')

nexttile
plot(xcoord,EsigtotalS1,xcoord,EpitotalS1)
legend('Total S1 energy','Pi Energies')
title('S1')

nexttile
plot(xcoord,EsigtotalT1,xcoord,EpitotalT1)
legend('Total T1 energy','Pi Energies')
title('T1')

nexttile
plot(xcoord,EsigtotalQ1,xcoord,EpitotalQ1)
legend('Total Q1 energy','Pi Energies')
title('Q1')

%All Energies
figure(6) %N.B. Here states are calculated based on orbital energies at a given
geometry, in fittings, states occupy same orbitals at every delta
plot(xcoord,EsigtotalS0rel,xcoord,EsigtotalS1rel,xcoord,EsigtotalT1rel,xcoord,Esigtot
alQ1rel)
legend('Total S0 energy','Total S1 energy','Total T1 energy','Total Q1 energy')
title('All state Energies')

ooph=transpose(OutOrbHomos); %Transposing orbital wavefunction arrays to fit the
table
oopl=transpose(OutOrbLumos);
iph=transpose(InOrbHomos);
ipl=transpose(InOrbLumos);
oophorbtable=table(xcoord,outhomoengs,ooph);
ooplorbtable=table(xcoord,outlumoengs,oopl);
```

```
iphorbtable=table(xcoord,inhomoengs,iph);
iplorbtable=table(xcoord,inlumoengs,ipl);

writetable(oophorbtable,'OutOfPlaneHOMOs.csv','Delimiter',',');
writetable(ooplorbtable,'OutOfPlaneLUMOs.csv','Delimiter',',');
writetable(iphorbtable,'InPlaneHOMOs.csv','Delimiter',',');
writetable(iplorbtable,'InPlaneLUMOs.csv','Delimiter',',');

disp('Variation of Total energy fitted to polynomial')
disp('Printing eigenvector error')

if Print_Frosts(1,1)=='N'
        disp('Frost diagram printing off')
elseif res>10
    disp('Too many iterations to print Frost diagrams - lower res')
else
    disp('Printing Frost diagrams')
figure(7);
    for i = 1:res+1 %Defining x value matrices for Frost diagram plotting
        for j = 1:n
            outtheta = asin((outallens(i,j)-a1)/(outallens(i,1)-a1)); %Defining theta
parameters
            intheta = asin((inallens(i,j)-a2)/(inallens(i,1)-a2));
            if mod(j,2)==0
            outfrostx(i,j)=real(((outallens(i,1)-a1))*(cos(outtheta))); %Calculating
x coordinates
            infrostx(i,j)=real(((inallens(i,1)-a2))*(cos(intheta)));
            else
            outfrostx(i,j)=real(-((outallens(i,1)-a1))*(cos(outtheta))); %Alternating
e values go either side of diagram
            infrostx(i,j)=real(-((inallens(i,1)-a2))*(cos(intheta)));
            end
            if showcircs(1,1)=='Y'
            outcirc=linspace(0,2*pi);
            xcout=real(outallens(i,1)-a1)*cos(outcirc);
            ycout=(outallens(i,1)-a1)*sin(outcirc)+a1;
            incirc=linspace(0,2*pi);
            xcin=(inallens(i,1)-a2)*cos(incirc);
            ycin=(inallens(i,1)-a2)*sin(incirc)+a2;
            else
            end
        end
    end
    frostplots=tiledlayout(2,res+1); % plotting frost diagrams as BLA changes
    frostplots.TileSpacing= 'none';
    for i=1:res+1
        nexttile
        if showcircs(1,1)=='Y'
     plot(outfrostx(i,:),outallens(i,:),".",xcout,ycout)
        else
            plot(outfrostx(i,:),outallens(i,:),".")
        end
     axis equal
    end
    for i=1:res+1
```

```
        nexttile
        if showcircs(1,1)=='Y'
        plot(infrostx(i,:),inallens(i,:),".",xcin,ycin)
        else
            plot(infrostx(i,:),inallens(i,:),".")
        end
        axis equal
    end
end

if pltmagints(1,1)== 'N'
    disp('Magnetic integral printing off')
else
    figure(8)
    tiledlayout(1,3)
    nexttile

plot(xcoord(2:res+1),outdiacont(2:res+1)+indiacont(2:res+1),xcoord(2:res+1),inparacon
t(2:res+1)+outparacont(2:res+1),xcoord(2:res+1),outtotcont(2:res+1)+intotcont(2:res+1
))
    legend('S0 diamagnetic contribution','S0 paramagnetic contribution','S0 total
magnetic effect  ')
    nexttile

plot(xcoord(2:res+1),outdiacont(2:res+1)+outtripdcont(2:res+1)+indiacont(2:res+1),xco
ord(2:res+1),inparacont(2:res+1),xcoord(2:res+1),inparacont(2:res+1)+outdiacont(2:res
+1)+outtripdcont(2:res+1)+indiacont(2:res+1))
    legend('T1 diamagnetic contribution','T1 paramagnetic contribution','T1 total')
    nexttile

plot(xcoord(2:res+1),outdiacont(2:res+1)+indiacont(2:res+1)+outsingdcont(2:res+1),xco
ord(2:res+1),insingpcont(2:res+1)+outsingpcont(2:res+1),xcoord(2:res+1),insingpcont(2
:res+1)+outsingpcont(2:res+1)+outdiacont(2:res+1)+indiacont(2:res+1)+outsingdcont(2:r
es+1))
    legend('S1 diamagnetic contribution','S1 paramagnetic contribution','S1 total')
end

    outfrosttab=table(outfrostx,outallens);
    infrosttab=table(infrostx,inallens);

    writetable(outfrosttab,'OutPlaneFrosts.csv','Delimiter',',')
    writetable(infrosttab,'InPlaneFrosts.csv','Delimiter',',')

disp('Script Finished')
```

GEOMETRIES

16

BLA=16

C	0.690385	-3.26183715	0.00
C	-0.690385	-3.26183715	0.00
C	1.8185091	-2.79477695	0.00
C	2.79477695	-1.8185091	0.00
C	3.26183715	-0.690385	0.00
C	-1.8185091	-2.79477695	0.00
C	-2.79477695	-1.8185091	0.00
C	-3.26183715	-0.690385	0.00
C	-3.26183715	0.690385	0.00
C	-2.79477695	1.8185091	0.00
C	-1.8185091	2.79477695	0.00
C	-0.690385	3.26183715	0.00
C	0.690385	3.26183715	0.00
C	1.8185091	2.79477695	0.00
C	3.26183715	0.690385	0.00
C	2.79477695	1.8185091	0.00

16

BLA=14.8

C	0.68732	-3.26146572	0.00
C	-0.68732	-3.26146572	0.00
C	1.82035928	-2.79231356	0.00
C	2.79231356	-1.82035928	0.00
C	3.26146572	-0.68732	0.00
C	-1.82035928	-2.79231356	0.00
C	-2.79231356	-1.82035928	0.00
C	-3.26146572	-0.68732	0.00

C	-3.26146572	0.68732	0.00
C	-2.79231356	1.82035928	0.00
C	-1.82035928	2.79231356	0.00
C	-0.68732	3.26146572	0.00
C	0.68732	3.26146572	0.00
C	1.82035928	2.79231356	0.00
C	3.26146572	0.68732	0.00
C	2.79231356	1.82035928	0.00

16

BLA=13.8

C	0.684255	-3.26109429	0.00
C	-0.684255	-3.26109429	0.00
C	1.82220946	-2.78985017	0.00
C	2.78985017	-1.82220946	0.00
C	3.26109429	-0.684255	0.00
C	-1.82220946	-2.78985017	0.00
C	-2.78985017	-1.82220946	0.00
C	-3.26109429	-0.684255	0.00
C	-3.26109429	0.684255	0.00
C	-2.78985017	1.82220946	0.00
C	-1.82220946	2.78985017	0.00
C	-0.684255	3.26109429	0.00
C	0.684255	3.26109429	0.00
C	1.82220946	2.78985017	0.00
C	3.26109429	0.684255	0.00
C	2.78985017	1.82220946	0.00

16

BLA=12.5

C	0.68119	-3.26072286	0.00

C	-0.68119	-3.26072286	0.00
C	1.82405964	-2.78738678	0.00
C	2.78738678	-1.82405964	0.00
C	3.26072286	-0.68119	0.00
C	-1.82405964	-2.78738678	0.00
C	-2.78738678	-1.82405964	0.00
C	-3.26072286	-0.68119	0.00
C	-3.26072286	0.68119	0.00
C	-2.78738678	1.82405964	0.00
C	-1.82405964	2.78738678	0.00
C	-0.68119	3.26072286	0.00
C	0.68119	3.26072286	0.00
C	1.82405964	2.78738678	0.00
C	3.26072286	0.68119	0.00
C	2.78738678	1.82405964	0.00

16

BLA=11.4

C	0.67812500	-3.26035143	0.00
C	-0.67812500	-3.26035143	0.00
C	1.82590982	-2.78492339	0.00
C	2.78492339	-1.82590982	0.00
C	3.26035143	-0.67812500	0.00
C	-1.82590982	-2.78492339	0.00
C	-2.78492339	-1.82590982	0.00
C	-3.26035143	-0.67812500	0.00
C	-3.26035143	0.67812500	0.00
C	-2.78492339	1.82590982	0.00
C	-1.82590982	2.78492339	0.00
C	-0.67812500	3.26035143	0.00

C	0.67812500	3.26035143	0.00
C	1.82590982	2.78492339	0.00
C	3.26035143	0.67812500	0.00
C	2.78492339	1.82590982	0.00

16

BLA=10.2

C	0.67506	-3.25998	0.00
C	-0.67506	-3.25998	0.00
C	1.82776	-2.78246	0.00
C	2.78246	-1.82776	0.00
C	3.25998	-0.67506	0.00
C	-1.82776	-2.78246	0.00
C	-2.78246	-1.82776	0.00
C	-3.25998	-0.67506	0.00
C	-3.25998	0.67506	0.00
C	-2.78246	1.82776	0.00
C	-1.82776	2.78246	0.00
C	-0.67506	3.25998	0.00
C	0.67506	3.25998	0.00
C	1.82776	2.78246	0.00
C	3.25998	0.67506	0.00
C	2.78246	1.82776	0.00

16

BLA=9.1

C	0.67202	-3.25956	0.00
C	-0.67202	-3.25956	0.00
C	1.82962	-2.78002	0.00

C	2.78002	-1.82962	0.00
C	3.25956	-0.67202	0.00
C	-1.82962	-2.78002	0.00
C	-2.78002	-1.82962	0.00
C	-3.25956	-0.67202	0.00
C	-3.25956	0.67202	0.00
C	-2.78002	1.82962	0.00
C	-1.82962	2.78002	0.00
C	-0.67202	3.25956	0.00
C	0.67202	3.25956	0.00
C	1.82962	2.78002	0.00
C	3.25956	0.67202	0.00
C	2.78002	1.82962	0.00

16

BLA=8

C	0.66898	-3.25914	0.00
C	-0.66898	-3.25914	0.00
C	1.83148	-2.77758	0.00
C	2.77758	-1.83148	0.00
C	3.25914	-0.66898	0.00
C	-1.83148	-2.77758	0.00
C	-2.77758	-1.83148	0.00
C	-3.25914	-0.66898	0.00
C	-3.25914	0.66898	0.00
C	-2.77758	1.83148	0.00
C	-1.83148	2.77758	0.00
C	-0.66898	3.25914	0.00
C	0.66898	3.25914	0.00
C	1.83148	2.77758	0.00

C	3.25914	0.66898	0.00
C	2.77758	1.83148	0.00

16

BLA=6.8

C	0.66594	-3.25872	0.00
C	-0.66594	-3.25872	0.00
C	1.83334	-2.77514	0.00
C	2.77514	-1.83334	0.00
C	3.25872	-0.66594	0.00
C	-1.83334	-2.77514	0.00
C	-2.77514	-1.83334	0.00
C	-3.25872	-0.66594	0.00
C	-3.25872	0.66594	0.00
C	-2.77514	1.83334	0.00
C	-1.83334	2.77514	0.00
C	-0.66594	3.25872	0.00
C	0.66594	3.25872	0.00
C	1.83334	2.77514	0.00
C	3.25872	0.66594	0.00
C	2.77514	1.83334	0.00

16

BLA=5.7

C	0.6629	-3.2583	0.00
C	-0.6629	-3.2583	0.00
C	1.8352	-2.7727	0.00
C	2.7727	-1.8352	0.00
C	3.2583	-0.6629	0.00
C	-1.8352	-2.7727	0.00
C	-2.7727	-1.8352	0.00

C	-3.2583	-0.6629	0.00
C	-3.2583	0.6629	0.00
C	-2.7727	1.8352	0.00
C	-1.8352	2.7727	0.00
C	-0.6629	3.2583	0.00
C	0.6629	3.2583	0.00
C	1.8352	2.7727	0.00
C	3.2583	0.6629	0.00
C	2.7727	1.8352	0.00

16

BLA=4.6

C	0.65986	-3.25788	0.00
C	-0.65986	-3.25788	0.00
C	1.83706	-2.77026	0.00
C	2.77026	-1.83706	0.00
C	3.25788	-0.65986	0.00
C	-1.83706	-2.77026	0.00
C	-2.77026	-1.83706	0.00
C	-3.25788	-0.65986	0.00
C	-3.25788	0.65986	0.00
C	-2.77026	1.83706	0.00
C	-1.83706	2.77026	0.00
C	-0.65986	3.25788	0.00
C	0.65986	3.25788	0.00
C	1.83706	2.77026	0.00
C	3.25788	0.65986	0.00
C	2.77026	1.83706	0.00

16

BLA=3.4

C	0.65682	-3.25746	0.00
C	-0.65682	-3.25746	0.00
C	1.83892	-2.76782	0.00
C	2.76782	-1.83892	0.00
C	3.25746	-0.65682	0.00
C	-1.83892	-2.76782	0.00
C	-2.76782	-1.83892	0.00
C	-3.25746	-0.65682	0.00
C	-3.25746	0.65682	0.00
C	-2.76782	1.83892	0.00
C	-1.83892	2.76782	0.00
C	-0.65682	3.25746	0.00
C	0.65682	3.25746	0.00
C	1.83892	2.76782	0.00
C	3.25746	0.65682	0.00
C	2.76782	1.83892	0.00

16

BLA=2.3

C	0.65378	-3.25704	0.00
C	-0.65378	-3.25704	0.00
C	1.84078	-2.76538	0.00
C	2.76538	-1.84078	0.00
C	3.25704	-0.65378	0.00
C	-1.84078	-2.76538	0.00
C	-2.76538	-1.84078	0.00
C	-3.25704	-0.65378	0.00
C	-3.25704	0.65378	0.00
C	-2.76538	1.84078	0.00
C	-1.84078	2.76538	0.00

C	-0.65378	3.25704	0.00
C	0.65378	3.25704	0.00
C	1.84078	2.76538	0.00
C	3.25704	0.65378	0.00
C	2.76538	1.84078	0.00

16

BLA=1.1

C	0.65074	-3.25662	0.00
C	-0.65074	-3.25662	0.00
C	1.84264	-2.76294	0.00
C	2.76294	-1.84264	0.00
C	3.25662	-0.65074	0.00
C	-1.84264	-2.76294	0.00
C	-2.76294	-1.84264	0.00
C	-3.25662	-0.65074	0.00
C	-3.25662	0.65074	0.00
C	-2.76294	1.84264	0.00
C	-1.84264	2.76294	0.00
C	-0.65074	3.25662	0.00
C	0.65074	3.25662	0.00
C	1.84264	2.76294	0.00
C	3.25662	0.65074	0.00
C	2.76294	1.84264	0.00

16

BLA=0

C	-2.34759451	2.34759451	0.00.00
C	-1.27050900	3.06728005	0.00.00
C	-3.06728005	1.27050900	0.00.00
C	-3.32000000	-0.00000000	0.00.00

C	-3.06728005	-1.27050900	0.00.00
C	-0.00000000	3.32000000	0.00.00
C	1.27050900	3.06728005	0.00.00
C	2.34759451	2.34759451	0.00.00
C	3.06728005	1.27050900	0.00.00
C	3.32000000	0.00000000	0.00.00
C	3.06728005	-1.27050900	0.00.00
C	2.34759451	-2.34759451	0.00.00
C	1.27050900	-3.06728005	0.00.00
C	0.00000000	-3.32000000	0.00.00
C	-2.34759451	-2.34759451	0.00.00
C	-1.27050900	-3.06728005	0.00.00

REFERENCES

(1) Gao Y.; Albrecht F.; Rončević I.; Ettedgui I.; Kumar P.; Scriven L.; Chistensen, K. E. M., S.; Righetti, L.; Rossmannek, M.; Tavernelli, I.; Anderson, H. L.; Gross, L. On-surface synthesis of a doubly anti-aromatic carbon allotrope: Cyclo[16]carbon. *ChemRxiv* **2023**.
(2) Neese, F.; Wennmohs, F.; Becker, U.; Riplinger, C. The orca quantum chemistry program package. *The Journal of Chemical Physics* **2020**, *152*, 224108. DOI: 10.1063/5.0004608
(3) *Gaussian 16 rev. C.01*; Wallingford, CT, 2016. (accessed.
(4) Aidas, K.; Angeli, C.; Bak, K. L.; Bakken, V.; Bast, R.; Boman, L.; Christiansen, O.; Cimiraglia, R.; Coriani, S.; Dahle, P.; et al. The dalton quantum chemistry program system. *WIREs Computational Molecular Science* **2014**, *4*, 269-284. DOI: https://doi.org/10.1002/wcms.1172
(5) Monaco, G.; Summa, F. F.; Zanasi, R. Program package for the calculation of origin-independent electron current density and derived magnetic properties in molecular systems. *Journal of Chemical Information and Modeling* **2021**, *61*, 270-283. DOI: 10.1021/acs.jcim.0c01136
(6) Sun, Q.; Zhang, X.; Banerjee, S.; Bao, P.; Barbry, M.; Blunt, N. S.; Bogdanov, N. A.; Booth, G. H.; Chen, J.; Cui, Z.-H.; et al. Recent developments in the p<scp>y</scp>scf program package. *The Journal of Chemical Physics* **2020**, *153*, 024109. DOI: 10.1063/5.0006074
(7) *Qiskit: An open-source framework for quantum computing*; 2019. 10.5281/zenodo.2562110 (accessed.
(8) Peruzzo, A.; McClean, J.; Shadbolt, P.; Yung, M.-H.; Zhou, X.-Q.; Love, P. J.; Aspuru-Guzik, A.; O'Brien, J. L. A variational eigenvalue solver on a photonic quantum processor. *Nature Communications* **2014**, *5*, 4213. DOI: 10.1038/ncomms5213
(9) Barkoutsos, P. K.; Gonthier, J. F.; Sokolov, I.; Moll, N.; Salis, G.; Fuhrer, A.; Ganzhorn, M.; Egger, D. J.; Troyer, M.; Mezzacapo, A.; et al. Quantum algorithms for electronic structure calculations: Particle-hole hamiltonian and optimized wave-function expansions. *Physical Review A* **2018**, *98*, 022322. DOI: 10.1103/PhysRevA.98.022322
(10) Spall, J. C. An overview of the simultaneous perturbation method for efficient optimization. *Johns Hopkins apl technical digest* **1998**, *19*, 482–492.
(11) Steiner, E.; Fowler, P. W. Patterns of ring currents in conjugated molecules: A few-electron model based on orbital contributions. *The Journal of Physical Chemistry A* **2001**, *105*, 9553-9562. DOI: 10.1021/jp011955m
(12) Fowler, P. W.; Steiner, E. Pseudo-π currents: Rapid and accurate visualisation of ring currents in conjugated hydrocarbons. *Chemical Physics Letters* **2002**, *364*, 259-266. DOI: https://doi.org/10.1016/S0009-2614(02)01244-7

Appendix: derivation for equation (5) Here, equation (5) from the main text is derived by transforming the basis set of p orbitals in a ring into a set of two cyclic wavefunctions.

Consider a cyclic ring consisting of n evenly spaced atoms, where n is an even number. The ring can be described as having n radial 'unit cells' of size a, where $a = \frac{2\pi}{n}$. The electron density's dependence on theta is therefore given by:

$$|\Psi(\theta + a)|^2 = |\Psi(\theta)|^2$$

$$\Psi(\theta + a) = \mu\Psi(\theta)$$

$$|\mu|^2 = 1$$

The cyclic nature of the ring implies that:

$$\Psi(\theta + na) = \Psi(\theta) = \mu^n\Psi(\theta)$$

$$\mu^n = 1 = e^{i2\pi k}, k \in \mathbb{Z}$$

$$\mu = e^{\frac{i2\pi k}{n}}$$

$$\Psi_k(\theta + a) = e^{\frac{i2\pi k}{n}}\Psi(\theta)$$

The simplest solution for $\Psi(\theta)$ is given by:

$$\Psi_k(\theta) = Ne^{\frac{i2\pi k}{na}\theta}$$

but as $a = \frac{2\pi}{n}$

$$\Psi_k(\theta) = Ne^{ik\theta}$$

These are the general solutions for the particle on a ring, where N is a normalisation factor. In order to account for the non-uniform density due to the atoms, we describe the basis $|\phi_m\rangle$, where m $\in \mathbb{N}, 0 \leq m \leq n-1$, which represent the p orbitals of each atom.

Each p orbital is located at ma radians around the ring, thus the wavefunctions can be written:

$$|\Psi_k\rangle = \sum_{m=0}^{n-1} Ne^{ikma}\,|\phi_m\rangle$$

As a is an arbitrary radial unit cell size, we can define a' as a unit cell containing two atoms, then $\frac{a'}{2} = a$, and $|\Psi_k\rangle$ can be written

$$|\Psi_k\rangle = \sum_{m=0}^{n-1} Ne^{ikm\frac{a'}{2}} |\phi_m\rangle$$

$$|\Psi_k\rangle = \sum_{m=0}^{\frac{n}{2}-1} Ne^{ik(2m)\frac{a'}{2}} |\phi_{2m}\rangle + \sum_{m=0}^{\frac{n}{2}-1} Ne^{ik(2m+1)\frac{a'}{2}} |\phi_{2m+1}\rangle$$

$$\sum_{m=0}^{\frac{n}{2}-1} N_A e^{ik(2m)\frac{a'}{2}} |\phi_{2m}\rangle = |\Psi_{k,A}\rangle$$

$$\sum_{m=0}^{\frac{n}{2}-1} N_B e^{ik(2m+1)\frac{a'}{2}} |\phi_{2m+1}\rangle = |\Psi_{k,B}\rangle$$

$|\Psi_{k,A}\rangle$ and $|\Psi_{k,B}\rangle$ represent the even and odd p orbitals in the ring, respectively. Trivially, it can be shown that $N_A = N_B = \sqrt{\frac{2}{n}}$. Perturbation of these wavefunctions to induce BLA corresponds to adding an offset of some small x, which for positive x, moves the even orbitals clockwise around the ring, and moves the odd orbitals anticlockwise around the ring, such that alternating bonds become longer and shorter

$$|\Psi_{k,A}\rangle = \sum_{m=0}^{\frac{n}{2}-1} \sqrt{\frac{2}{n}} e^{ik(2m)\frac{a'}{2}+ix} |\phi_{2m}\rangle$$

$$|\Psi_{k,B}\rangle = \sum_{m=0}^{\frac{n}{2}-1} \sqrt{\frac{2}{n}} e^{ik(2m+1)\frac{a'}{2}-ix} |\phi_{2m+1}\rangle$$

The energies of this system can be described by the equation

$$\underline{\underline{H}}\underline{c} = E\underline{\underline{S}}\underline{c}$$

$$\begin{pmatrix} H_{AA} & H_{AB} \\ H_{BA} & H_{BB} \end{pmatrix} \begin{pmatrix} \Psi_{k,A} \\ \Psi_{k,B} \end{pmatrix} = E_k \begin{pmatrix} 1 & 0 \\ 0 & 1 \end{pmatrix} \begin{pmatrix} \Psi_{k,A} \\ \Psi_{k,B} \end{pmatrix}$$

$$\begin{pmatrix} H_{AA} - E_k & H_{AB} \\ H_{BA} & H_{BB} - E_k \end{pmatrix} \begin{pmatrix} \Psi_{k,A} \\ \Psi_{k,B} \end{pmatrix} = 0$$

$$\begin{vmatrix} H_{AA} - E_k & H_{AB} \\ H_{BA} & H_{BB} - E_k \end{vmatrix} = 0$$

$$(H_{AA} - E_k)(H_{BB} - E_k) - H_{AB}H_{BA} = 0$$

Defining the Hamiltonian matrix elements between two p orbitals (where m and l are under modulo n to maintain the cyclic boundary condition)

$$\langle \phi_{2m}| H |\phi_{2l+1}\rangle = \begin{cases} \beta(1+\delta) & : m = l \\ \beta(1-\delta) & : m = l+1 \\ 0 & : \text{otherwise} \end{cases}$$

$$\langle \phi_{2m+1}| H |\phi_{2l}\rangle = \begin{cases} \beta(1+\delta) & : m = l \\ \beta(1-\delta) & : m = l-1 \\ 0 & : \text{otherwise} \end{cases}$$

$$\langle \phi_{2m}| H |\phi_{2l}\rangle = \begin{cases} \alpha & : m = l \\ 0 & : \text{otherwise} \end{cases}$$

$$\langle \phi_{2m+1}| H |\phi_{2l+1}\rangle = \begin{cases} \alpha & : m = l \\ 0 & : \text{otherwise} \end{cases}$$

Then calculating each matrix element:

$$H_{AA} = \langle \Psi_{k,A}| H |\Psi_{k,A}\rangle = \sum_{m=0}^{\frac{n}{2}-1} \sum_{l=0}^{\frac{n}{2}-1} \frac{2}{n} e^{-ik(2m)\frac{a'}{2}-ix} e^{ik(2l)\frac{a'}{2}+ix} \langle \phi_{2m}| H |\phi_{2l}\rangle$$

$$= \sum_{m=0}^{\frac{n}{2}-1} \frac{2}{n}\alpha = \alpha$$

$$H_{BB} = \langle \Psi_{k,B}| H |\Psi_{k,B}\rangle = \sum_{m=0}^{\frac{n}{2}-1} \sum_{l=0}^{\frac{n}{2}-1} \frac{2}{n} e^{-ik(2m+1)\frac{a'}{2}+ix} e^{ik(2l+1)\frac{a'}{2}-ix} \langle \phi_{2m+1}| H |\phi_{2l+1}\rangle$$

$$= \sum_{m=0}^{\frac{n}{2}-1} \frac{2}{n}\alpha = \alpha$$

$$H_{AB} = \langle \Psi_{k,A}| H |\Psi_{k,B}\rangle = \sum_{m=0}^{\frac{n}{2}-1} \sum_{l=0}^{\frac{n}{2}-1} \frac{2}{n} e^{-ik(2m)\frac{a'}{2}-ix} e^{+ik(2l+1)\frac{a'}{2}-ix} \langle \phi_{2m}| H |\phi_{2l+1}\rangle$$

$$= \sum_{m=0}^{\frac{n}{2}-1} \frac{2}{n}\beta(1+\delta)e^{ik\frac{a'}{2}-i2x} + \beta(1-\delta)e^{-ik\frac{a'}{2}-i2x}$$

$$= \beta(1+\delta)e^{ik\frac{a'}{2}-i2x} + \beta(1-\delta)e^{-ik\frac{a'}{2}-i2x}$$

$$H_{BA} = \langle \Psi_{k,B}| H |\Psi_{k,A}\rangle = \sum_{m=0}^{\frac{n}{2}-1} \sum_{l=0}^{\frac{n}{2}-1} \frac{2}{n} e^{-ik(2m+1)\frac{a'}{2}+ix} e^{+ik(2l)\frac{a'}{2}+ix} \langle \phi_{2m+1}| H |\phi_{2l}\rangle$$

$$= \sum_{m=0}^{\frac{n}{2}-1} \frac{2}{n}\beta(1+\delta)e^{-ik\frac{a'}{2}+i2x} + \beta(1-\delta)e^{ik\frac{a'}{2}+i2x}$$

$$= \beta(1+\delta)e^{-ik\frac{a'}{2}+i2x} + \beta(1-\delta)e^{ik\frac{a'}{2}+i2x}$$

Then substituting these expressions into:

$$(H_{AA} - E_k)(H_{BB} - E_k) - H_{AB}H_{BA} = 0$$

$$(\alpha - E_k)^2 - (\beta(1+\delta)e^{-ik\frac{a'}{2}+i2x} + \beta(1-\delta)e^{ik\frac{a'}{2}+i2x})(\beta(1+\delta)e^{ik\frac{a'}{2}-i2x} + \beta(1-\delta)e^{-ik\frac{a'}{2}-i2x}) = 0$$

$$(\alpha - E_k)^2 - (\beta^2(1+\delta)^2 + \beta^2(1-\delta^2) + \beta^2(1+\delta)(1-\delta)(e^{ik\frac{a'}{2}-i2x}e^{ik\frac{a'}{2}+i2x} + e^{-ik\frac{a'}{2}+i2x}e^{-ik\frac{a'}{2}-i2x})) = 0$$

$$(\alpha - E_k)^2 - (\beta^2(1+\delta)^2 + \beta^2(1-\delta)^2 + \beta^2(1+\delta)(1-\delta)(e^{ika'} + e^{-ika'})) = 0$$

$$(\alpha - E_k)^2 - \beta^2(2 + 2\delta^2 + (1-\delta^2)(2\cos ka')) = 0$$

$$(\alpha - E_k)^2 = 2\beta^2(1 + \delta^2 + (1-\delta^2)\cos ka')$$

Then as $a' = \frac{4\pi}{n}$ we finally obtain:

$$E_k = \alpha \pm \sqrt{2}\beta\sqrt{(1 + \delta^2 + (1-\delta^2)\cos\frac{4\pi k}{n})}$$
